# Development of the project-level Women’s Empowerment in Agriculture Index (pro-WEAI)

**DOI:** 10.1016/j.worlddev.2019.06.018

**Published:** 2019-10

**Authors:** Hazel Malapit, Agnes Quisumbing, Ruth Meinzen-Dick, Greg Seymour, Elena M. Martinez, Jessica Heckert, Deborah Rubin, Ana Vaz, Kathryn M. Yount, Gender Agriculture Assets Project Phase 2 (GAAP2) Study Team

**Affiliations:** a International Food Policy Research Institute, Washington, DC, USA; b Cultural Practice, LLC, USA; c Oxford Poverty and Human Development Initiative, United Kingdom; d Emory University, USA

**Keywords:** Agency, Agricultural development, Multidimensional measurement, Gender equality, Women’s empowerment

## Abstract

With growing commitment to women’s empowerment by agricultural development agencies, sound methods and indicators to measure women’s empowerment are needed to learn which types of projects or project-implementation strategies do and do not work to empower women. The Women’s Empowerment in Agriculture Index (WEAI), which has been widely used, requires adaptation to meet the need for monitoring projects and assessing their impacts.

In this paper, the authors describe the adaptation and validation of a project-level WEAI (or pro-WEAI) that agricultural development projects can use to identify key areas of women’s (and men’s) disempowerment, design appropriate strategies to address identified deficiencies, and monitor project outcomes related to women’s empowerment. The 12 pro-WEAI indicators are mapped to three domains: intrinsic agency (power within), instrumental agency (power to), and collective agency (power with). A gender parity index compares the empowerment scores of men and women in the same household. The authors describe the development of pro-WEAI, including: (1) pro-WEAI’s distinctiveness from other versions of the WEAI; (2) the process of piloting pro-WEAI in 13 agricultural development projects during the Gender, Agriculture, and Assets Project, phase 2 (GAAP2); (3) analysis of quantitative data from the GAAP2 projects, including intrahousehold patterns of empowerment/disempowerment; and (4) a summary of the findings from the qualitative work exploring concepts of women’s empowerment in the project sites. The paper concludes with a discussion of lessons learned from pro-WEAI and possibilities for further development of empowerment metrics.

## Highlights

• The pro-WEAI is a new tool designed to meet projects’ impact assessment needs.
• Projects identified and field-tested pro-WEAI indicators using mixed methods.
• Pro-WEAI is mapped to 3 domains: intrinsic, instrumental, and collective agency.
• Pro-WEAI is decomposable into sub-indices, indicators, and by population subgroup.
• Women are more disempowered and have a higher intensity of disempowerment than men.

## 1 Introduction

Valid and comprehensive measures of gender equality and women’s empowerment are essential to monitor progress toward achieving Sustainable Development Goal (SDG) 5. Women’s empowerment and gender equality are important in their own right to women and girls and are linked with other SDGs, such as eliminating poverty (SDG 1), achieving zero hunger and malnutrition (SDG 2), and good health and well-being for women and children (SDG 3) (Cunningham et al., 2015, Heckert et al., 2019, Malapit et al., 2015, Ruel et al., 2018, Sraboni et al., 2014).

Many agricultural development interventions aim to empower women alongside goals to improve agricultural productivity and income; reduce poverty, hunger, and undernutrition; and improve health outcomes. Despite this growing commitment to gender equality and women’s empowerment among funders and implementers of agricultural development projects and the proliferation of women’s empowerment measures, consistent approaches for measuring women’s empowerment in agricultural development projects are lacking. Appropriate metrics are needed to assess whether these projects are achieving their goals.

Many analyses of women’s empowerment have drawn on a typology of power that is rooted in the seminal works of Freire (1968) on freedom and Lukes (1974) on power and articulated with respect to gender and women’s empowerment by Rowlands, 1995, Rowlands, 1997. This typology juxtaposes the notion of dominating or exerting “power over” others, with generative forms of empowerment, including “power within” (involving self-respect, self-efficacy, and an awareness of rights),^2^ “power to” (enacting personal goals), and “power with” (acting collectively toward shared interests) (see, also, Ibrahim & Alkire, 2007). This framing is common not only in the academic literature, but also in guidance for development programming (e.g., Luttrell & Quiroz, 2009) because of its practical implications.

Most indices of women’s empowerment have been measured and reported at the national level because they rely on administrative or aggregate data, and thus focus on gender equality, rather than women’s empowerment. Alkire et al. (2013) reviewed some of these indices, such as the Gender Gap Index (World Economic Forum [2018] and prior years), Gender Development Index (GDI), and Gender Inequality Index (GII) (UNDP, 2018). These indices measure gender inequalities in a broad set of domains but do not measure women’s empowerment comprehensively or rely on only indirect proxies, such as women’s age, schooling attainment, and share of parliamentary seats. Moreover, because these indices rely on national-level aggregate data, they cannot be decomposed by region or population subgroups. Several authors have recognized the limitations of using existing measures of gender equality to measure women’s empowerment (Alkire, 2005, Alsop et al., 2005, Kishor and Subaiya, 2008, Narayan, 2005, cited in Alkire et al., 2013, Yount et al., 2016).

Recent measures of empowerment, such as the Women’s Empowerment in Agriculture Index (WEAI) (Alkire et al., 2013), operationalize Kabeer’s (1999) definition of empowerment as the process by which people expand their ability to make strategic life choices, particularly in contexts in which this ability had been denied to them. In Kabeer’s definition, the ability to exercise choice encompasses three dimensions: resources (defined to include not only access but also future claims to material, human, and social resources), agency (including processes of decision-making, negotiation, and even deception and manipulation), and achievements (well-being outcomes).

Filling a niche unaddressed by existing metrics, the WEAI measures women’s empowerment in the agricultural sector directly through a focus on women’s agency using individual-level data collected from male and female household members in a household survey designed for this purpose. The WEAI is an aggregate index, reported at the country or sub-national level and comprised of two sub-indices. The first sub-index assesses the degree to which respondents are empowered in five domains of empowerment (5DE) in agriculture, namely, decisions about agricultural production, access to and decision-making power about productive resources, control of use of income, leadership in the community, and time allocation (Alkire et al., 2013). It reflects the percentage of women and men who are empowered and, among those who are not, the percentage of domains in which they achieve a pre-defined threshold for adequacy in empowerment. The second sub-index, the Gender Parity Index (GPI), measures gender parity. The GPI reflects the percentage of women who are empowered or whose achievements are at least as high as the men in their households. For those households that have not achieved gender parity, the GPI shows the empowerment gap that needs to be closed for women to reach the same level of empowerment as men in their households (Alkire et al., 2013).

The WEAI’s focus on women’s empowerment in the agricultural sector is important, given that agriculture remains the basis for the livelihoods of most rural people in low- and middle-income countries. Originally, the WEAI was intended as a monitoring and evaluation (M&E) tool for the US Government’s Feed the Future (FTF) initiative to track changes in women’s empowerment in agriculture over time and assess differences across countries, regions, and population subgroups. The WEAI was suited to this purpose, given its broad applicability and transparent design, and its original domains were chosen based on the areas that United States Agency for International Development (USAID) aimed to affect directly in FTF programming.

More than a single number, the WEAI provides an “information platform” (Alkire, 2018) for measuring women’s empowerment in agriculture. It includes multiple sub-indices and indicators that provide complementary, yet unique, pieces of information. As an aggregate, headline figure, the WEAI not only provides an overall measure of women’s empowerment that is decomposable at multiple levels depending on the data’s sample design, but is also decomposable into its component sub-indices or by indicator. Further, because the WEAI uses data from both male and female respondents, one can make direct comparisons between men and women in the same household and separately diagnose the aggregate sources of disempowerment for men and women. Such gender comparisons are not possible using other available empowerment measures (e.g., based on Demographic and Health Surveys), which do not typically cover both men and women. The transparency of the WEAI stems directly from its counting-based measurement approach, for which the definitions, thresholds, and weights of each indicator are explicitly defined (Alkire et al., 2015).^3^

Since its launch, at least 86 organizations in 53 countries (as of June 2019) have fielded the WEAI, often adapting it for their own use.^4^ Some adaptations were made to shorten interview time, but at the cost of removing key aspects of the index. Other modifications capture aspects of women’s empowerment that were not included in the WEAI. However, the ad hoc adaptations jeopardized comparability to the original index, limiting the ability of users to learn from each other and synthesize lessons across different settings.

Meanwhile, research on the measurement of women’s empowerment has flourished. A survey by O’Hara and Clement (2018) uses WEAI data and qualitative data from Nepal on local meanings of empowerment to suggest the importance of adding critical consciousness to the measures of agency. Several survey-based efforts are underway using different methodologies from the WEAI to measure particular aspects of women’s livelihoods. For example, the International Livestock Research Institute (ILRI) and Emory University, recognizing the importance of livestock to rural communities in East Africa, developed the Women’s Empowerment in Livestock Index (WELI) to explore how livestock is related to and supports women’s empowerment and the health and nutrition of women and children (Galiè et al., 2019). The WELI focuses on key areas of livestock production, such as animal health, breeding, and feeding; as well as the use of livestock products, such as animal-source-food processing and marketing.^5^ Similarly, the Women’s Empowerment in Nutrition Index (WENI) aims to capture nutritional empowerment, or “the process by which individuals acquire the capacity to be well fed and healthy” (Narayanan, Fontana, Lentz, & Kulkarni, 2019). This process entails gaining access to, and control over, key resources, including *intakes of food* that are adequate and nutritious; *knowledge* about nutritional and health practices; and *support* from family and other institutions in securing and maintaining an adequate diet and health. These resources may enhance women’s agency, specifically their influence in decisions over the production, acquisition/procurement, and distribution of food. The authors rely on the heuristic WENI grid in which empowerment (resources, agency, and achievements) is measured in the domains of health, nutrition, and institutions, to identify areas of disempowerment that may influence poor nutritional outcomes. The authors focus on the nutritional empowerment of women, such that the nutritional outcomes of interest are those of women themselves, rather than of their children (Narayanan et al., 2019).

Outside the agricultural sector, other approaches for developing measures of women’s empowerment have included exploratory factor analysis, and more recently, novel applications of item response theory (IRT) and structural equation modeling (Crandall et al., 2015, Cheong et al., 2017, Miedema et al., 2018, Yount et al., 2014, Yount et al., 2016). Such methods are especially useful for identifying survey questions that are valid measures of multifaceted constructs, like women’s agency. To be valid, such measures need to be conceptually sound and empirically (or psychometrically) “comparable” across groups and over time. Using these methods, Yount and colleagues have identified three indices of women’s ***intrinsic agency***. The first index—women’s perceived right to bodily integrity—uses attitudinal questions about intimate partner violence (IPV) against women that are psychometrically comparable across genders (Yount et al., 2014), age-at-marriage groups (Yount et al., 2016), and countries (Miedema et al., 2018). The second index—women’s perceived self-efficacy—validates the generalized self-efficacy scale in young Qatari women (Crandall et al., 2015). The third index—women’s perceived social and economic rights—uses attitudinal questions derived from qualitative research that are psychometrically comparable across Qatari and non-Qatari women (Yount, James-Hawkins and Abdul-Rahim nd).

Other analyses by Yount and colleagues have identified two indices for women’s ***instrumental agency***. The first index—women’s influence in household decisions—uses survey questions that capture a woman’s influence in decisions about her own earnings, her husband’s earnings, large or daily household purchases, seeking medical treatment, and visits to family and friends; psychometrically, these questions are valid at the national level in several countries (Yount et al., 2016, Miedema et al., 2018) and are comparable across age-at-marriage subgroups (Yount et al., 2016), countries (Miedema et al., 2018), and time (Cheong et al., 2017). The second index—women’s freedom of movement—uses survey questions that capture the ability of women to visit important venues outside the home; psychometrically, these questions also are valid at the national level (Yount et al., 2016), and are comparable across age-at-marriage subgroups (Yount et al., 2016) and over time (Cheong et al., 2017). The project-level WEAI (pro-WEAI) team is now leveraging similar methodologies to examine the psychometric properties of pro-WEAI (Yount et al., 2019), and will in the future aim to construct a validated, shorter version of pro-WEAI that measures the same concepts as the original for national- and program-level monitoring.

The original WEAI was developed for population-based monitoring of the FTF initiative. Since then, both researchers and implementing organizations have undertaken broad and diverse adaptations of the WEAI, aiming to develop indices that focus on aspects of agricultural livelihoods not covered by the original WEAI. Demand is clearly high for a standardized and validated measure of women’s empowerment that is useful for agricultural development projects to assess the impact of their projects on women’s empowerment, and to focus on outcomes that could change over the typical two- to five-year project cycle. This need is especially acute for projects that aim to empower women, not just reach or benefit them (Johnson et al., 2018). Outcome indicators must also detect potential unintended consequences that could result from women’s participation in such projects, such as backlash from men as a result of projects that specifically target and/or empower women (World Bank/FAO/IFAD, 2008) and increased constraints on women’s time which may, in turn, negatively affect women’s own health and nutrition as well as the health and nutrition of their children (Ruel et al., 2018).

To address this demand, pro-WEAI builds on the WEAI, but with more explicit links to empowerment theory and adapts it for use as a metric for measuring the impact of agriculture development projects on women’s empowerment, as well as a diagnostic tool for tailoring such programs to specific settings. Following this introduction, the methodology section describes how pro-WEAI was developed collaboratively with 13 agricultural development projects in Africa and South Asia as part of the Gender, Agriculture, and Assets Project, Phase 2 (GAAP2), and how the quantitative and qualitative data were collected to develop and validate pro-WEAI. The next section provides an overview of the structure of pro-WEAI, including the definition of domains and indicators and the computation of the index, drawing from the qualitative research related to local understandings of empowerment. This section is followed by a presentation of the quantitative data on pro-WEAI from five participating projects for which complete data on all indicators are available, including robustness checks. The paper concludes by discussing what we are learning from pro-WEAI and possibilities for further development of empowerment metrics.

## 2 Methodology

To develop an index that would be useful for projects, we worked with a portfolio of agricultural development projects that had explicit women’s empowerment goals to identify what they desired in a measurement tool and to learn what works best, in terms of measurement and implementation, under different conditions (Table 1). The revisions of existing survey instruments and the development of new ones occurred through a process that engaged the literature using and critiquing the WEAI, and that drew on the expert knowledge of program implementers and researchers who have conducted quantitative and qualitative studies on women’s empowerment. These individuals collaboratively engaged in the design of the survey instrument by proposing content to pilot. The project teams field-tested the new materials, using qualitative and quantitative methods. Baseline quantitative data were then shared with the pro-WEAI team for analysis, validation, and creation of a draft pro-WEAI. Feedback on the draft index was elicited from the participant projects and expert stakeholders in the research and development communities.^6^

**Table 1:** Projects in the GAAP2 portfolio.

Project name	Partner organization(s)	Country	Commodity focus	Project outcome	Status of qualitative work
**Agriculture, Nutrition, and Gender Linkages (**ANGeL**)**	Bangladesh Ministry of Agriculture and International Food Policy Research Institute (IFPRI)	Bangladesh	Crops	Nutrition	Qualitative work completed (around process evaluation); not included in paper
**Bangladesh Agriculture Value Chains (**AVC**)**	Development Alternatives Incorporated (DAI) and IFPRI	Bangladesh	Crops	Nutrition and income	Qualitative work completed; included in paper
**Food and Agricultural Approaches to Reducing Malnutrition (**FAARM**)**	Helen Keller International and University of Heidelberg	Bangladesh	Crops and livestock	Nutrition	Qualitative work currently underway or recently completed; not included in paper
**Targeting and Realigning Agriculture to Improve Nutrition (**TRAIN**)**	BRAC and IFPRI	Bangladesh	Crops	Nutrition	Qualitative work currently underway or recently completed; not included in paper
**Building resilience of vulnerable communities in Burkina Faso (**Grameen**)**	Grameen Foundation and Brigham Young University	Burkina Faso	Crops and livestock	Nutrition and income	Qualitative work completed; included in paper
**Integrated poultry value chain and nutrition intervention (**SE LEVER**)**	Agribusiness Systems International, AfricSante, and IFPRI	Burkina Faso	Livestock	Nutrition and income	Qualitative work currently underway or recently completed; not included in paper
**UN Joint Programme on accelerating progress towards the economic empowerment of rural women in Ethiopia (**JP-RWEE**)**	Food and Agriculture Organization of the United Nations, International Fund for Agricultural Development, United National Entity for Gender Equity and the Empowerment of Women, World Food Programme	Ethiopia	Crops and livestock	Nutrition and income	Qualitative work completed; included in paper
**Small-scale irrigation and women’s empowerment in northern Ghana (**iDE**)**	International Development Enterprises (iDE) and IFPRI	Ghana	Crops	Nutrition and income	Qualitative work completed; included in paper
**Women Improving Nutrition through Group-based Strategies (**WINGS**)**	Professional Assistance for Development Action (PRADAN) and IFPRI	India	Crops and livestock	Nutrition	Qualitative work currently underway or recently completed; not included in paper
**MoreMilk: Making the most of milk (**MoreMilk**)**	International Livestock Research Institute, IFPRI, International Institute for Environment and Development, and Emory University	Kenya	Livestock	Nutrition and income	Qualitative work completed; included in paper ^**^Only study to complete qualitative work before quantitative baseline
**Deploying improved vegetable technologies to overcome malnutrition and poverty in Mali (**WorldVeg**)**	World Vegetable Center	Mali	Crops	Nutrition and income	Qualitative work completed; included in paper
**Empowerment, Resilience, and Livestock Transfers (**Heifer**)**	Heifer Project International, Montana State University, University of Georgia, IFPRI, and Nepā School of Social Sciences and Humanities	Nepal	Livestock	Nutrition and income	Qualitative work completed; included in paper
**Evaluation of women’s food security program for impoverished Maasai households (**Maisha Bora**)**	Savannas Forever, Trias Tanzania, and University of Minnesota	Tanzania	Livestock	Nutrition and income	Qualitative work completed (Round 1); included in paper

All the projects in the portfolio have multiple focal outcomes. In addition to women’s empowerment, all projects aim to improve nutrition outcomes, and some projects also aim to improve incomes. All projects are collecting some combination of core and supplemental pro-WEAI modules as part of their impact evaluations, and each project will assess empowerment impacts alongside other outcomes in the context of their interventions. In this paper, we focus only on the pro-WEAI to describe the development of the tool. Individual project teams will assess the impact of their interventions on empowerment and other outcomes when their endline surveys are completed.

### 2.1 Quantitative methods

Baseline data collection using the pilot pro-WEAI questionnaire occurred between April 2016 and June 2018. The WEAI and pro-WEAI differ in the choice of survey respondents. In the WEAI, the primary male and female adults in each household were interviewed; in pro-WEAI, the respondents were the intended beneficiary(ies) of the intervention. For example, the female beneficiary and her spouse or other primary male decision-maker in the household, or the equivalent in the control group.^7^ Since many of the GAAP2 projects are targeted to women, we assume (for simplicity) that the eligible participant is a woman. Differences in project designs and sampling strategies may result in systematically different distributions of age and other characteristics for women and men in the project samples. These differences should be considered when interpreting pro-WEAI results.

Owing to changes made to pro-WEAI following the inception workshop, five projects collected only a partial version of the pro-WEAI questionnaire. Three projects did not collect or collected modified versions of the questionnaire at baseline.^8^ Five projects in the portfolio were used to validate this version of the pro-WEAI: ANGeL, AVC, SE LEVER, TRAIN and WorldVeg (Ahmed et al., 2018, De Brauw et al., 2019, Gelli et al., 2017).

### 2.2 Qualitative methods

Although pro-WEAI is computed based on survey data, qualitative research was an important part of the index’s development to gain a better understanding of the conditions of poverty and women’s disempowerment, to assess the salience of the pro-WEAI domains in local contexts, and to understand the linkages between project interventions and women’s empowerment outcomes. Prior qualitative work done to develop the original WEAI and other qualitative research done in the project areas (e.g. Pradhan, Meinzen-Dick, & Theis, 2018) provided important insights. As with the survey, the qualitative methods for pro-WEAI were developed through a participatory process with the project teams (for details on the methods, see Meinzen-Dick et al., 2019). The qualitative protocols included guidelines for the following: review of project documents; a community profile; a seasonality calendar; key informant interviews with project staff and with traders and marketers; focus group discussions on local meanings of empowerment; and semi-structured life history interviews with project participants and participants from control groups. The qualitative teams adapted these protocols to align with project-specific priorities.

The qualitative findings described in this paper are based on data collected by eight of the 13 projects between November 2016 and February 2018, which were available for analysis when developing pro-WEAI. To help develop the questionnaire, ideally, the qualitative studies would have preceded the surveys. However, project schedules precluded this except in one case (MoreMilk), and one other case (Heifer) had previous qualitative research on many of the indicators of empowerment. Despite scheduling limitations, the team leading the qualitative research interacted regularly with the index development team and made explicit efforts to bring insights from the qualitative work in constructing the index. We drew on prior qualitative work on the WEAI as well as the current studies in shaping the content of the survey modules, formulating some of the indicators and determining the thresholds for adequacy and empowerment, and understanding the correlations between empowerment and other indicators. These processes are discussed in the presentation of the domains and indicators, and in more detail in Meinzen-Dick et al., 2019.

## 3 The project-level Women’s Empowerment in Agriculture Index (pro-WEAI)

### 3.1 Domains and indicators of pro-WEAI

Both the WEAI and pro-WEAI are rooted in Kabeer, 1999, Kabeer, 2005 framework of empowerment, which describes empowerment as a process of change on the interrelated dimensions of resources, agency, and achievements, and focuses on measuring agency, or the ability of individuals to make strategic choices. Both indices focus on the agency dimension for both conceptual and practical reasons. Conceptually, one could argue that agency is a more *direct* measure of empowerment, compared with resources or achievements, both of which could exist even in situations where women are extremely disempowered. Another consideration is the need to reduce complexity by prioritizing which empowerment dimensions are missing in standard data collection, and those for which methodologies are least developed. Information about key resources (including various aspects of human and social capital), and achievements (such as productivity, incomes, or nutrition) is typically collected in impact assessment surveys. Although current methods for collecting information on resources (e.g., Doss et al., 2008, GAAP, 2014) and on achievements (outcomes) can be improved, these methodologies are better developed than methodologies for measuring agency, for which few standardized measures exist that are validated widely across contexts and over time. Adding indicators of agency further allows for study of how resources, agency, and achievements interact.

Whereas the original WEAI has five domains of empowerment with ten indicators organized thematically and is aligned with FTF programming priorities, pro-WEAI has 12 indicators mapped to three domains: intrinsic agency (power within), instrumental agency (power to), and collective agency (power with). These three aspects of agency reflect the generative types of power described above (Rowlands, 1997, Ibrahim and Alkire, 2007) and are present in the earlier WEAI, although not explicitly. These theoretical links are strengthened in the pro-WEAI.

Based on the consistent negative perceptions of coercive agency (power over) that were revealed in the qualitative research, that type of agency is not included in the index. This exclusion is consistent with the observation by Rowlands (1997:11):

When power is defined as 'power over', then if women gain power it will be at men's expense. It is easy to see why the notion of women becoming empowered is seen as inherently threatening, the assumption being that there will be some kind of reversal of relationships, and men will not only lose power but also face the possibility of having power wielded over them by women.

The rejection of ‘power over’ as empowerment is reflected in our treatment of attitudes about intimate partner violence (IPV), to be discussed below.

Participating projects identified what they thought were essential and measurable indicators for assessing whether their projects’ strategies to empower women are working. Projects viewed many of the existing WEAI indicators, such as group membership, as important, given that many projects use groups as a strategy for building social capital and delivering training to beneficiaries. They also suggested many new indicators, such as those reflecting intrinsic agency. For example, projects were concerned about potential backlash against women as their incomes improved, which may be captured by attitudes toward IPV. Some interventions used strategies directed toward increasing women’s self-confidence or improving intrahousehold harmony. These aspects are reflected in new indicators such as self-efficacy and respect among household members.

Table 2 presents full definitions for the pro-WEAI indicators and, if the indicator was previously included in the WEAI, how the pro-WEAI indicator differs. The four indicators of intrinsic agency include autonomy in income, self-efficacy, attitudes about IPV against women,^9^ and respect among household members. The six indicators of instrumental agency include input into productive decisions, ownership of land and other assets, control over use of income, access to and decisions on financial services, work balance, and visiting important locations. Collective agency is comprised of group membership and membership in influential groups. Seven out of the 12 indicators in pro-WEAI are adapted from the original WEAI indicators, 10and five indicators are new (attitudes about IPV against women, self-efficacy, respect among household members, visiting important locations, membership in influential groups) and stem from topics that the projects themselves suggested. Each indicator is equally weighted, and a person is defined as empowered if she or he is empowered in at least nine of 12, or 75 percent, of the indicators.

**Table 2:** Pro-WEAI indicators, definitions of adequacy, and comparison to the original WEAI.

Indicator ^A^	Definition of adequacy	Difference compared to original WEAI
*Intrinsic Agency*		
Autonomy in income	More motivated by own values than by coercion or fear of others’ disapproval: *Relative Autonomy Index*^B^ score>=1 RAI score is calculated by summing responses to the three vignettes about a person’s motivation for how they use income generated from agricultural and non-agricultural activities (yes = 1; no = 0), using the following weighting scheme: 0 for vignette 1 (no alternative), −2 for vignette 2 (external motivation), −1 for vignette 3 (introjected motivation), and +3 for vignette 4 (autonomous motivation)	Based on “Autonomy in production” indicator in the WEAI but now focuses exclusively on the use of income generated from agricultural and non-agricultural activities and uses a new vignette-based survey instrument.
Self-efficacy	“Agree” or greater on average with self-efficacy questions: *New General Self-Efficacy Scale*^C^ score>=32	Not included in the WEAI
Attitudes about intimate partner violence against women	Believes husband is NOT justified in hitting or beating his wife in all 5 scenarios: ^D^ 1) She goes out without telling him 2) She neglects the children 3) She argues with him 4) She refuses to have sex with him 5) She burns the food	Not included in the WEAI
Respect among household members	Meets ALL of the following conditions related to their spouse, the other respondent, or another household member: 1) Respondent respects relation (MOST of the time) AND 2) Relation respects respondent (MOST of the time) AND 3) Respondent trusts relation (MOST of the time) AND 4) Respondent is comfortable disagreeing with relation (MOST of the time)	Not included in the WEAI

*Instrumental Agency*		
Input in productive decisions	Meets at least ONE of the following conditions for ALL of the agricultural activities they participate in 1) Makes related decision solely, 2) Makes the decision jointly and has at least some input into the decisions 3) Feels could make decision if wanted to (to at least a MEDIUM extent)	Included in the WEAI, but now uses a stricter adequacy cut-off
Ownership of land and other assets	Owns, either solely or jointly, at least ONE of the following: 1) At least THREE small assets (poultry, nonmechanized equipment, or small consumer durables) 2) At least TWO large assets 3) Land	Included in the WEAI, but now uses a stricter adequacy cut-off
Access to and decisions on financial services	Meets at least ONE of the following conditions: 1) Belongs to a household that used a source of credit in the past year AND participated in at least ONE sole or joint decision about it 2) Belongs to a household that did not use credit in the past year but could have if wanted to from at least ONE source 3) Has access, solely or jointly, to a financial account	Based on “Access to and decisions on credit” indicator in the WEAI, but now includes access to financial accounts
Control over use of income	Has input in decisions related to how to use BOTH income and output from ALL of the agricultural activities they participate in AND has input in decisions related to income from ALL non-agricultural activities they participate in, unless no decision was made	Included in the WEAI, but now uses a stricter adequacy cut-off
Work balance	Works less than 10.5 h per day: Workload = time spent in primary activity + (1/2) time spent in childcare as a secondary activity	Similar to ‘Workload” indicator in the WEAI but restricts the measurement of secondary activities to a single activity: childcare.
Visiting important locations	Meets at least ONE of the following conditions: 1) Visits at least TWO locations at least ONCE PER WEEK of [city, market, family/relative], or 2) Visits least ONE location at least ONCE PER MONTH of [health facility, public meeting]	Not included in the WEAI

*Collective Agency*		
Group membership	Active member of at least ONE group	Same as in the WEAI
Membership in influential groups	Active member of at least ONE group that can influence the community to at least a MEDIUM extent	Not included in the WEAI

**Notes:**^A^ All indicators are equally weighted (1/12) in the pro-WEAI.

^B^ The Relative Autonomy Index (RAI), based on self-determination theory, is a measure of internal and external motivations that determine person’s decisions (Ryan & Deci, 2000). The text for vignettes 1–4 can be found in Appendix C, module G8(A).

^C^ The New General Self-efficacy Scale (NGSE) is a validated scale to measure self-efficacy, or a person’s capabilities and ability to reach their goals (Chen, Gully, & Eden, 2001). The questions can be found in Appendix C, module G8(B).

These scenarios are based on previously validated items from the Demographic and Health Surveys (Yount et al., 2014).

The WEAI and pro-WEAI rely heavily on instrumental agency indicators, comprised mainly of decision-making questions. Decision-making questions are often used in surveys, and span many different aspects (e.g., production, assets, credit, etc.), so these questions have been tested and used more widely than have indicators on intrinsic and collective agency. The reliance on instrumental agency implies that households with only female decision-makers are more likely to be identified as empowered by default, which is a known limitation of WEAI (Alkire et al., 2013). While a number of aspects of instrumental agency are well established in the theoretical literature, we had a smaller pool of candidate indicators to draw on for measuring intrinsic and collective agency in developing pro-WEAI.^11^

These indicators align with existing theoretical domains and qualitative research in local contexts. The focus groups and individual interviews often described an empowered person as having resources or achievements, rather than agency, because the former are easier to conceptualize and to observe. Resources could be tangible, such as livestock among pastoralist societies (the Maasai in Tanzania or Fulani in Mali), or less tangible, such as education (in Ethiopia, Mali, Nepal, and Bangladesh [AVC]) or connections to the outside (in Ghana and Nepal). Expressions of empowerment in terms of achievements often focused on having sufficient financial resources, manifested in good personal appearance and providing good food, clothing, housing and education for family members (Meinzen-Dick et al., 2019).

Expressions of empowerment as agency also emerged, phrased in terms of taking care of oneself, or being strong or able (e.g., Maisha Bora case of Maasai in Tanzania and JP-RWEE case in Ethiopia). The MoreMilk study in Kenya was illustrative: empowered milk traders were described as business-minded, making smart decisions, being good with customers, and maintaining hygienic standards for handling milk—a mix of intrinsic and instrumental agency. In almost all cases, women’s empowerment was associated with helping other people, reflecting a pursuit of common goals or collective agency. Such notions of collective agency tend to be grounded in the family but may extend to others in the community. These expressions of collective agency go beyond “power with” and might be better described as “power for others.”

Among other indicators of intrinsic agency, self-efficacy was not often articulated in qualitative research, but there was considerable discussion of self-efficacy related to IPV perpetrated against women. A focus group participant in the Maisha Bora study in Tanzania indicated how violence can affect self-efficacy:

I’m worried to make any other decisions because I might be beaten by my husband and he tells me that I’m nothing and can’t do anything that can bring fruits to this family (Krause, James, McCarthy, & Bellemare, 2018:31).

Thus, internalizing the acceptability of IPV affects women’s intrinsic agency.

Women often described intrahousehold harmony as important to them, both for its intrinsic value and because harmonious relations with husbands and in-laws would enable women to do more, including having greater capacity to move freely, attend group meetings, and earn income.

Focus groups in the Ghana, Kenya, and Burkina Faso qualitative studies cited decision-making as an aspect of empowerment, but independent decision-making was not necessarily desired. In the Bangladesh AVC study, women as well as men said that it was not good for women to make decisions independently. In Ethiopia, Ghana, and Mali, participants talked about the importance of women at least consulting their husbands as a sign of respect, or to maintain intrahousehold harmony. In the Ghana case, women privately expressed a desire for more input into decisions, but not having sole decision-making, in case something went wrong. Consistent with these aspirations for decision-making, pro-WEAI considers either sole or joint decision-making as empowering. We also include the potential for the respondent to be involved in decisions *if they wanted to* as empowering, because women do not necessarily want to be included in every decision, and therefore should not be counted as disempowered if they have the option to participate, but do not, out of their own choice.

In pro-WEAI, we consider ownership of land and other assets to be an indicator of instrumental agency, rather than a measure of resources in Kabeer’s framework because this indicator measures self-reported ownership, rather than externally-recognized rights to resources. For example, in the Maisha Bora study among Maasai in Tanzania, 96 percent of men and 65 percent of women report owning land either solely or jointly, although they rarely have any documentation of these land rights (Krause et al., 2018). Qualitative research on the pro-WEAI has repeatedly shown that agency is involved in realizing rights over resources (Meinzen-Dick et al., 2019). For example, qualitative research on control over assets in the study areas in Nepal illustrates the various types of agency women employ. Speaking of personal property (e.g., goats, small assets) classified as “pewa,” women often spoke of “doing pewa” in an active sense, rather than more passively “having pewa” (Pradhan et al., 2018). Hence, we argue that the act of claiming ownership over an asset is itself a reflection of agency. Prior quantitative analysis of the WEAI also supports this argument by revealing a high-degree of correlation between self-reported ownership of an asset and a bundle of property rights associated with control over the asset, which were included in previous WEAI surveys (Malapit et al., 2017).

Other instrumental agency indicators, such as access to financial services was discussed as empowering in the context of savings and loan groups, and formal bank accounts for milk traders in the MoreMilk case. Work balance was not mentioned explicitly as an aspect of empowerment, but excessive workloads were discussed as limiting women’s ability to do many other things, including attending group meetings or earning income. The discussions of freedom of movement showed the extent of restrictions on women’s ability to leave the homestead owing to gender norms and lack of time, as well as the importance of mobility to enable women to attend group meetings and earn income.

The discussions of group membership gave clear examples of how participation in groups could be empowering through new access to information, resources, and connections with others. Depending on the context, the types of groups that play this role could vary, including not only formal producers’ organizations and savings and loan associations, but also funeral societies and other self-help groups, labor exchange groups, or civic and religious groups.^12^ The survey therefore asks about a wide range of types of formal and informal groups, and the group membership indicator counts membership in any of these as empowering, but the influential groups indicator is based on the respondent’s assessment of the group’s influence in the community. Thus, group membership and membership in influential groups are suitable indicators of collective agency, although they may not go far enough to capture local definitions of empowerment as the ability to help others.

### 3.2 Computation of the index

Each respondent in the pro-WEAI is classified as either adequate (=1) or inadequate (=0) in a given indicator by comparing their responses to the survey questions with a given threshold (Table 2). A respondent’s empowerment score is simply the weighted average of her/his adequacy scores in the 12 indicators (all weighted 1/12). If her/his score is 75% or higher, or if s/he is adequate in nine out of 12 indicators, then s/he is classified as empowered. Conversely, if her/his score is below 75%, or if s/he is inadequate in 4 or more indicators, then s/he is classified as disempowered. These individual level scores are then aggregated to construct pro-WEAI.

Pro-WEAI, similar to the original WEAI, is calculated as the weighted mean of two sub-indices: the Three Domains of Empowerment Index (3DE), with a weight of 90 percent, and the GPI, with a weight of 10 percent. The 3DE measures women’s empowerment across three domains: **intrinsic agency** (power within), **instrumental agency** (power to), and **collective agency** (power with). The GPI compares the empowerment scores of the eligible individual and her spouse, or the male respondent, in each household. The choice of weights for the two sub-indices follows the original WEAI, placing greater emphasis on the 3DE while still recognizing the importance of gender equality as an aspect of empowerment. Improvements in either the 3DE or GPI will increase pro-WEAI scores. While the aggregate pro-WEAI index, 3DE for women, 3DE for men, and GPI are all useful ways to summarize empowerment at the project level, we recommend interpreting these high-level indexes together with the sub-indicators, and sub-components. The decomposability of the index allows the user to disaggregate the drivers of change, and examine how women’s and men’s empowerment scores are contributing to it. Details on how the individual indicators are combined to form the pro-WEAI index are presented in Appendix B.

## 4 Results

### 4.1 Quantitative data and pro-WEAI results

Table 3 presents basic demographic information for the combined sample of five projects for which complete data on all indicators are available. Most respondents were between the ages of 16 and 45 years, and female respondents were younger than male respondents, on average. Most respondents had either never attended school or had attended only primary school. Nearly all respondents were married at the time of the survey.

**Table 3:** Demographic characteristics of respondents.

Variable	Percent of respondents	
	Female	Male
*Age group*		
16–25	32.1	6.3
26–45	57.5	62.2
46–65	9.9	28.1
>65	0.2	3.3
Missing	0.3	0.2

*Education*		
Never attended school	44.9	46.1
Less than primary	13.9	19.3
Primary	33.4	24.5
Secondary	7.0	7.7
Undergraduate or higher	0.0	0.1
Missing	0.9	2.3

*Marital status*		
Married	98.8	97.7
Unmarried (never married)	0.2	1.6
Unmarried (previously married)	0.8	0.5
Missing	0.2	0.2

**Source:** Baseline data from ANGeL (N = 7523), AVC (N = 1000), SE LEVER (N = 3342), TRAIN (N = 9823), and WorldVeg (N = 1408).

**Note:** Weighted by inverse project sample size.

The aggregate pro-WEAI score for women in the pilot baseline sample, weighted by inverse project sample size, is 0.59. This figure is the weighted average of the 3DE score for women, 0.57, and the GPI score, 0.77 (Table 4). Sixteen percent of women and 43 percent of men in this sample are empowered according to pro-WEAI. Of those women who are disempowered, the mean adequacy score is 0.49; these women achieve adequacy in an average of 49 percent of the indicators. Of men who are identified as disempowered, the mean adequacy score was 0.59, indicating that these men achieve adequacy in an average of 59 percent of the indicators. The GPI score is 0.77, and 30 percent of households achieved gender parity. The average empowerment gap between women who do not achieve gender parity and the men in their households is 33 percent.

**Table 4:** Pro-WEAI results.

Indicator	Women	Men
Number of observations	11,513	10,689
**3DE score**	**0.57**	**0.76**
Disempowerment score (1 — 3DE)	0.43	0.24
% achieving empowerment	16%	43%
% not achieving empowerment	84%	57%
Mean adequacy score for not yet empowered	0.49	0.59
Mean disempowerment score (1 — adequacy) for not yet empowered	0.51	0.41

Number of dual-adult households	10,689	
**Gender Parity Index (GPI)**	**0.77**	
% achieving gender parity	30%	
% not achieving gender parity	70%	
Average empowerment gap	0.33	
**Pro-WEAI score**	**0.59**	

**Source:** Baseline data from ANGeL (N = 7523), AVC (N = 1000), SE LEVER (N = 3342), TRAIN (N = 9823), and WorldVeg (N = 1408).

**Note:** Weighted by inverse project sample size. Respondents with missing indicators are dropped from the sample.

**Fig. 1:**
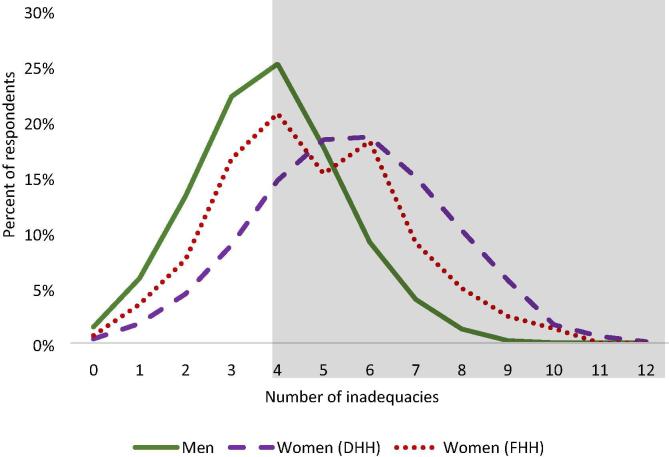
Distribution of inadequacies. **Source:** Baseline data from ANGeL (N = 7523), AVC (N = 1000), SE LEVER (N = 3342), TRAIN (N = 9,823), and WorldVeg (N = 1408). **Notes:** Shaded box indicates disempowered respondents, i.e., those who are inadequate in four or more indicators. Weighted by inverse project sample size. DHH = dual-adult household that includes both a male and female adult. FHH = female-adult-only household that includes a female adult but no male adult.

The 3DE score represents the achievements of women in the sample across the 12 indicators of empowerment in pro-WEAI. The 3DE considers the number of women who are disempowered and the intensity of their disempowerment, or the number of indicators in which these women are *not* adequately empowered. Fig. 1 compares the number of inadequacies among men and women. Overall, men have fewer inadequacies than women. The individuals in the shaded box in Fig. 1, who are inadequate in four or more indicators, are disempowered. Within the shaded box, the distributions of inadequacies for women in female-adult-only households (FHH) and women in dual-adult households (DHH) lie to the right of men’s distribution of inadequacies. More women than men are disempowered, and disempowered women have more inadequacies, on average, than disempowered men. In other words, women experience a higher intensity of disempowerment than do men. Intensity of disempowerment is the average proportion of indicators in which respondents are not adequately empowered.

When analyzing the pro-WEAI results, comparing the uncensored and censored headcount ratio is useful (Table 5). The censored headcount ratio is the proportion of respondents who are disempowered *and* inadequate in a given indicator. The uncensored headcount ratio, on the other hand, is the proportion of respondents who are inadequate in a given indicator, regardless of their empowerment status.^13^ A higher proportion of women compared to men are inadequate across all 12 indicators. The gap in adequacy between women and men is largest for work balance and ability to visit important locations. Most women (84%) are disempowered, so the uncensored and censored headcount ratios for women are similar. For men, the uncensored and censored headcount ratios are similar only for input in productive decisions and ownership of land and other assets, which suggests that most men who are inadequate in these indicators are disempowered. There is a large difference between the uncensored and censored headcount ratios for men for group membership and membership in influential groups, meaning that a large proportion of men are inadequate in these indicators but not disempowered.

**Table 5:** Headcount ratios and relative contributions of each indicator to disempowerment.

Indicator	Uncensored headcount ratio (%)		Censored headcount ratio (%)		Proportional contribution to disempowerment (%)	
	Men	Women	Men	Women	Men	Women
*Intrinsic agency*						
Autonomy in income	38.6	41.7	26.5	39.3	9.3	7.5
Self-efficacy	36.8	49.3	28.6	46.5	9.9	8.9
Attitudes about intimate partner violence against women	34.6	49.1	25.5	45.6	8.9	8.8
Respect among household members	25.0	38.4	17.9	36.0	6.2	6.9

*Instrumental agency*						
Input in productive decisions	7.4	18.4	6.8	18.2	2.4	3.5
Ownership of land and other assets	1.1	21.6	1.0	20.3	0.3	3.9
Access to and decisions on financial services	24.4	40.4	18.6	39.1	6.5	7.5
Control over use of income	13.4	33.2	11.1	32.4	3.9	6.2
Work balance	33.5	61.5	24.2	55.5	8.4	10.7
Ability to visit important locations	31.8	59.5	25.4	53.4	8.9	10.2

*Collective agency*						
Group membership	63.7	64.8	48.9	61.6	17.0	11.8
Membership in influential groups	71.5	79.1	52.6	73.2	18.2	14.0

**Source:** Baseline data from ANGeL (N = 7523), AVC (N = 1000), SE LEVER (N = 3342), TRAIN (N = 9823), and WorldVeg (N = 1408).

**Notes:** The censored headcount ratio reflects the percent of respondents who are both disempowered and inadequate in the indicator. Uncensored headcount ratio reflects the percent of respondents who are inadequate in the indicator. Weighted by inverse project sample size.

The proportional contribution of each indicator to disempowerment reflects how much each indicator contributes to disempowerment among respondents who have not achieved empowerment. It is calculated as the censored headcount ratio for a given indicator divided by the total empowerment score, multiplied by the indicator’s weight times 100.

**Fig. 2:**
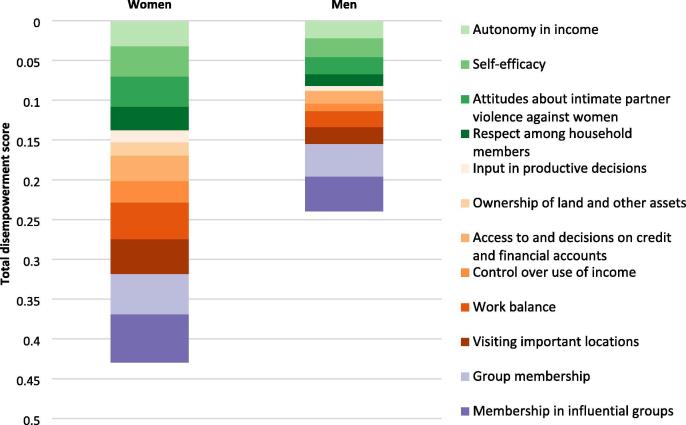
Contributions of each indicator to disempowerment. **Source:** Baseline data from ANGeL (N = 7523), AVC (N = 1000), SE LEVER (N = 3342), TRAIN (N = 9823), and WorldVeg (N = 1408). **Note:** Weighted by inverse project sample size.

Fig. 2 depicts the absolute contribution of each indicator to disempowerment for men and women in the sample. The overall depth of each bar shows the total disempowerment score (1- 3DE), and the different colored bars within show the absolute contribution of each indicator to disempowerment.^14^ Overall, women are more disempowered than men. The largest contributors to disempowerment for women and men are group membership and membership in influential groups. Visiting important locations, work balance, self-efficacy, attitudes about IPV against women, and autonomy in income also are large contributors to disempowerment for women. The similarities and differences between women’s and men’s disempowerment profiles point to opportunities for interventions to close empowerment gaps by addressing them in program design.

#### 4.1.1 Intrahousehold patterns of empowerment

We use data from individuals living in DHH to examine intrahousehold patterns of empowerment (Table 6). In most DHH (72%), the man is adequate in more indicators than the woman; the woman is adequate in more indicators than the man in 16 percent of households; and the man and the woman are equally adequate in 12 percent of households. On average, the male respondent is adequate in 15 percent more indicators (approximately two indicators) than the female respondent in the same household.

**Table 6:** Intrahousehold patterns of empowerment.

	% of dual-adult households
Male adequacy score > female adequacy score	72.0
Female adequacy score > male adequacy score	16.2
Female adequacy score = male adequacy score	11.8
Only male is empowered	35.3
Only female is empowered	8.1
Both male and female are empowered	7.4
Neither male nor female are empowered	49.2

**Source:** Baseline data from ANGeL (N = 7523), AVC (N = 1000), SE LEVER (N = 3342), TRAIN (N = 9823), and WorldVeg (N = 1408).

**Note:** Weighted by inverse project sample size.

In the overall sample, most men (57%) and women (84%) are disempowered. In about half of DHH, neither the man nor the woman achieved empowerment. In about a third of households, only the man is empowered.

#### 4.1.2 Decomposition of the 3DE score by age group

The 3DE is decomposable at any level for which the dataset is representative. For example, in the pro-WEAI results above, the 3DE is decomposed by gender. The analogous 5DE score from the original WEAI is often decomposed by sub-regions or other groups within a country. For an impact evaluation, projects may find it useful to decompose the 3DE by other categories, such as demographic or treatment groups. Here, we present an example of decomposition by the woman’s age group.

First, projects can compare the aggregate pro-WEAI scores between groups (Table 7). In this example, the pro-WEAI, 3DE, and GPI scores are all highest among women aged 26–45 years compared to younger and older women, meaning that women in this middle age group are more empowered and have greater parity with the men in their households.

**Table 7:** Pro-WEAI results by age group.

Indicator	Age 16–25		Age 26–45		Age 46+	
	Women	Men	Women	Men	Women	Men
Number of observations	5148	4786	5862	5290	444	399
**3DE score**	**0.58**	**0.76**	**0.63**	**0.77**	**0.58**	**0.74**
Disempowerment score (1 – 3DE)	0.42	0.24	0.37	0.23	0.42	0.26
% achieving empowerment	0.18	0.40	0.23	0.44	0.17	0.40
% not achieving empowerment	0.82	0.60	0.77	0.56	0.83	0.60
Mean 3DE score for not yet empowered	0.49	0.59	0.52	0.60	0.49	0.58
Mean disempowerment score (1 – 3DE)	0.51	0.41	0.48	0.40	0.51	0.42
Number of dual-adult households	4786		5290		399	
**Gender Parity Index (GPI)**	**0.77**		**0.82**		**0.79**	
% achieving gender parity	0.32		0.39		0.36	
% not achieving gender parity	0.68		0.61		0.64	
Average empowerment gap	0.34		0.29		0.33	
**Pro-WEAI score**	**0.59**		**0.65**		**0.60**	

**Source:** Baseline data from ANGeL (N = 7523), AVC (N = 1000), SE LEVER (N = 3342), TRAIN (N = 9823), and WorldVeg (N = 1408).

**Note:** Weighted by inverse project sample size.

**Fig. 3:**
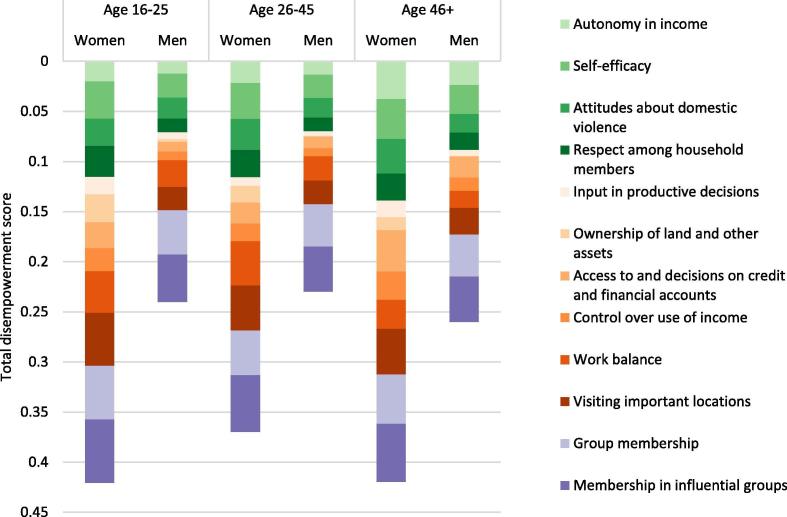
Contributors to disempowerment by age group. **Source:** Baseline data from ANGeL (N = 7523), AVC (N = 1000), SE LEVER (N = 3342), TRAIN (N = 9823), and WorldVeg (N = 1408). **Note:** Weighted by inverse project sample size.

Projects can also compare the contributions to disempowerment of each indicator between groups. In this example, the largest contributors to disempowerment for all three age groups are group membership and membership in influential groups. Ownership of land and other assets is a much larger contributor to disempowerment for women than men in all three age groups. Some contributors varied between age groups. Work balance was a larger contributor to disempowerment for women aged 16–25 and 26–45 compared to older women; control over use of income and autonomy in income were larger contributors to disempowerment for women aged 46 and older compared to younger women (Fig. 3).

#### 4.1.3 Robustness tests

##### 4.1.3.1 Nonresponse rates

To estimate pro-WEAI, responses are necessary for every indicator for each individual in the sample. Nonresponse, or missing data, occurs when the respondent has not answered the specific survey questions needed to calculate the indicator. For example, a respondent must be an active member of at least one community group to be considered adequate in group membership. If the respondent has not answered the survey questions about whether they participated in groups, their response is considered missing for that indicator.^15^

Across the five projects that collected the complete survey instrument, nonresponse rates are generally low (Table 8). Except for two indicators, access to and decisions on financial services and work balance, the proportion of missing data among men and women in DHHs is below 1 percent. For these two indicators, nonresponse rates, although higher, are still low, ranging from 1.5 to 2.8 percent for financial services, and 2.6 to 3.2 percent for work balance. The relatively higher nonresponse rates for these questions could be related to people’s reluctance to answer questions about finances as well as difficulty in recalling time spent in various activities, which is required for the work balance indicator. Overall, 96 percent of respondents in these projects answered all of the questions needed to compute all 12 indicators. We observe a relatively higher proportion of nonresponse in FHHs. Notably, in 17 percent of FHHs, there was only one adult living in the household. Hence, women in these households were not able to answer the questions necessary for the respect among household members indicator.

**Table 8:** Percent nonresponse for each pro-WEAI indicator.

Indicator	Men (dual-adult)	Women (dual-adult)	Women (female-only)
*Intrinsic agency*			
Autonomy in income	0.3	0.3	1.5
Self-efficacy	0.2	0.0	0.8
Attitudes about intimate partner violence against women	0.1	0.1	0.8
Respect among household members	0.8	0.9	17.3

*Instrumental agency*			
Input in productive decisions	0.1	0.1	0.0
Ownership of land and other assets	0.1	0.0	0.0
Access to and decisions on financial services	2.8	1.5	1.8
Control over use of income	0.1	0.1	0.0
Work balance	3.2	2.6	3.2
Ability to visit important locations	0.3	0.0	0.0

*Collective agency*			
Group membership	0.1	0.0	0.0
Membership in influential groups	0.1	0.0	0.0

**Source:** Baseline data from ANGeL (N = 7523), AVC (N = 1000), SE LEVER (N = 3342), TRAIN (N = 9823), and WorldVeg (N = 1408).

**Note:** Weighted by inverse project sample size.

##### 4.1.3.2 Association analysis

Next, we consider pairwise associations between pro-WEAI indicators using Cramer’s V. A high pairwise correlation could result in a greater than intended implicit weight being assigned to an indicator pair, which would need to be considered and justified. Most of the 12 pro-WEAI indicators are weakly correlated with each other (Cramer’s V <0.30) (Table 9). There is a moderate correlation between input in productive decisions and control over use of income (V = 0.502), and there is a strong correlation between group membership and membership in influential groups (V = 0.728) which is expected because the latter is derived from the former.

**Table 9:** Association (Cramer’s V) between pro-WEAI indicators.

	Autonomy in income	Self-efficacy	Attitudes about intimate partner violence against women	Respect among household members	Input in productive decisions	Ownership of land and other assets
*Intrinsic agency*						
Autonomy in income	1.000					
Self-efficacy	0.072	1.000				
Attitudes about intimate partner violence against women	0.051	0.062	1.000			
Respect among household members	0.068	0.135	0.081	1.000		

*Instrumental agency*						
Input in productive decisions	0.111	0.083	0.008	0.044	1.000	
Ownership of land and other assets	−0.016	0.112	0.005	0.090	0.089	1.000
Access to and decisions on financial services	0.115	0.086	0.030	0.013	0.173	0.052
Control over use of income	0.091	0.104	0.032	0.094	0.502	0.099
Work balance	−0.014	−0.011	0.018	0.008	−0.020	0.028
Ability to visit important locations	−0.061	0.103	0.006	0.047	0.029	0.217
*Collective agency*						
Group membership	0.000	0.003	−0.047	−0.033	0.042	0.017
Membership in influential groups	−0.025	0.020	−0.039	0.005	0.023	0.076
	Access to and decisions on financial services	Control over use of income	Work balance	Ability to visit important locations	Group membership	Membership in influential groups

*Instrumental agency*						
Access to and decisions on financial services	1.000					
Control over use of income	0.122	1.000				
Work balance	−0.010	0.033	1.000			
Ability to visit important locations	0.007	0.023	0.021	1.000		
*Collective agency*						
Group membership	0.058	0.039	0.015	0.073	1.000	
Membership in influential groups	−0.002	0.063	0.051	0.095	0.728	1.000

**Source:** Baseline data from ANGeL (N = 7523), AVC (N = 1000), SE LEVER (N = 3342), TRAIN (N = 9823), and WorldVeg (N = 1408).

In the case of input into productive decisions, control over use of income, and influence in community groups, this high correlation may be a consequence of survey design, because the questions underlying these indicators are posed in sequence within the same survey module. Yount et al. (2019) explore this issue using IRT methods and find a similar association. Follow-up cognitive testing is planned to investigate this issue.

The correlation is expected in the case of group membership and membership in influential groups, given the definition of the indicators: to be adequate in membership in influential groups, a person must be a member of a group (i.e., adequate in group membership). Defining the indicators in this way was deliberate, designed to increase the implicit weight of collective agency within pro-WEAI, given the relative lack of collective agency indicators in the index, compared to intrinsic and instrumental agency indicators. However, this structure also makes this component sensitive to systematic measurement error, such as under-reporting of group membership. Work already is underway to design and validate new indicators of collective agency for inclusion in future revisions of pro-WEAI.

An alternative way to examine the relationship between indicators is redundancy. Redundancy between two indicators, A and B, is defined by Alkire et al. (2015) as the proportion of respondents inadequate in indicator A who also are inadequate in indicator B, where A is the indicator in which fewer respondents are inadequate. Thus, 61 percent of respondents inadequate in autonomy in income also are inadequate in self-efficacy (Table 10). Overall, there is high redundancy among the 12 pro-WEAI indicators. Given that we do not observe a similarly high degree of correlation between all the indicators, we do not interpret high redundancy as problematic from a measurement perspective but as evidence that inadequacies tend to be clustered. Indeed, at this stage of instrument development and adaptation, redundancy allows us to adapt indicators so they provide complementary information and, if well-supported, will help streamline pro-WEAI for different purposes.

**Table 10:** Redundancy between pro-WEAI indicators.

	Autonomy in income	Self-efficacy	Attitudes about intimate partner violence against women	Respect among household members	Input in productive decisions	Ownership of land and other assets
*Intrinsic agency*						
Autonomy in income	1.000					
Self-efficacy	0.599	1.000				
Attitudes about intimate partner violence against women	0.636	0.595	1.000			
Respect among household members	0.693	0.730	0.707	1.000		

*Instrumental agency*						
Input in productive decisions	0.889	0.885	0.881	0.871	1.000	
Ownership of land and other assets	0.871	0.915	0.877	0.893	0.901	1.000
Access to and decisions on financial services	0.672	0.663	0.656	0.667	0.876	0.874
Control over use of income	0.793	0.788	0.809	0.771	0.962	0.899
Work balance	0.553	0.560	0.585	0.649	0.850	0.865
Ability to visit important locations	0.556	0.613	0.566	0.695	0.892	0.939

*Collective agency*						
Group membership	0.577	0.581	0.572	0.642	0.896	0.899
Membership in influential groups	0.557	0.593	0.566	0.643	0.921	0.934

	Access to and decisions on financial services	Control over use of income	Work balance	Ability to visit important locations	Group membership	Membership in influential groups

*Instrumental agency*						
Access to and decisions on financial services	1.000					
Control over use of income	0.786	1.000				
Work balance	0.646	0.777	1.000			
Ability to visit important locations	0.662	0.772	0.536	1.000		

*Collective agency*						
Group membership	0.706	0.781	0.528	0.609	1.000	
Membership in influential groups	0.694	0.807	0.563	0.648	0.999	1.000

**Source:** Baseline data from ANGeL (N = 7523), AVC (N = 1000), SE LEVER (N = 3342), TRAIN (N = 9823), and WorldVeg (N = 1408).

##### 4.1.3.3 Rank robustness

In pro-WEAI, the 12 indicators are weighted equally, and a respondent is considered empowered if s/he is adequate in at least 75 percent, or nine of 12, of the indicators. Rank robustness analysis was performed, following Alkire et al. (2015), to assess whether changing indicator weights or empowerment cut-offs affects the comparison of pro-WEAI results between projects.

**Fig. 4:**
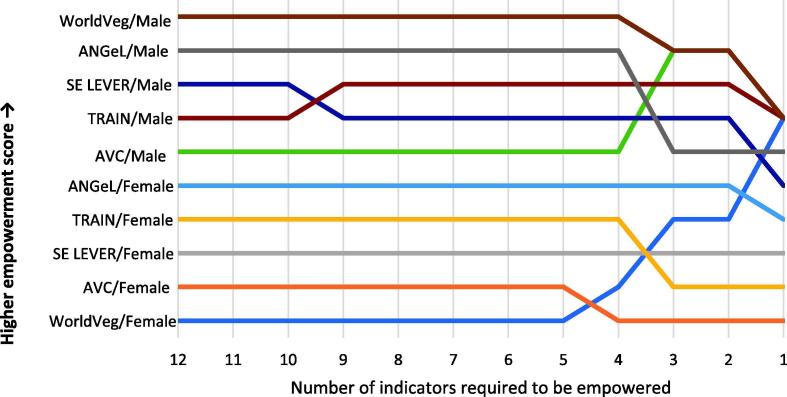
Rank comparison of 3DE scores by project and gender for different empowerment cut-offs. **Source:** Baseline data from ANGeL (N = 7523), AVC (N = 1000), SE LEVER (N = 3342), TRAIN (N = 9823), and WorldVeg (N = 1408). **Notes:** 3DE scores ranked from highest to lowest. Spearman’s rho = 1.000; Kendall’s tau b = 1.000. Weighted by inverse project sample size.

First, we rank projects’ 3DE scores for different empowerment cut-offs, where a higher ranking indicates a higher 3DE score (Fig. 4). We consider the full spectrum of possible cut-offs. The ranking is the same for empowerment cut-offs between five and nine indicators and registers few changes for the wider range between four and 11 indicators. Significant changes in the ranking occurs for cutoffs below four. However, we disregard these changes in rankings for cutoffs below four as it would be difficult to justify on theoretical grounds identifying an individual, adequate in no more than 25 percent of the indicators, as empowered. Thus, we find that changing the empowerment cut-off has little meaningful impact on comparison across projects.

Next, we compare how projects rank by 3DE score for different indicator weighting schemes (Table 11). We consider two weighting schemes: equal weighting by indicators (the chosen scheme), in which each of the 12 indicators is given a 1/12 weight, and equal weighting by domain, in which each of the three theoretical domains (intrinsic, instrumental, and collective agency) is given equal weight, and the indicators within each domain are evenly weighted. While there is some difference in the rankings of projects across the two weighting schemes, the rank correlation coefficients are positive and high (Spearman’s rho = 0.903, Kendall’s tau b = 0.822), indicating high concordance between weighting schemes.

**Table 11:** Rank of 3DE scores by project and gender for different weighting schemes.

Project/Gender	Equally weighted by indicator	Equally weighted by domain
WorldVeg/Female	1	1
AVC/Female	2	2
SE LEVER/Female	3	3
TRAIN/Female	4	4
**ANGeL/Female**	**5**	**6**
**AVC/Male**	**6**	**7**
**SE LEVER/Male**	**7**	**9**
**TRAIN/Male**	**8**	**5**
**ANGeL/Male**	**9**	**8**
WorldVeg/Male	10	10

**Source:** Baseline data from ANGeL (N = 7523), AVC (N = 1000), SE LEVER (N = 3342), TRAIN (N = 9823), and WorldVeg (N = 1408).

**Notes:** 3DE scores ranked from highest to lowest (1 = highest score; 10 = lowest score). Spearman’s rho = 0.903; Kendall’s tau b = 0.822. Groups where ranking differs in **bold**. Weighted by inverse project sample size.

## 5 Concluding remarks

### 5.1 Lessons from pro-WEAI development

Recognizing that women’s empowerment is important in its own right and important for achieving other outcomes such as income, health, and nutrition of women and their families, pro-WEAI was developed as a metric that captured aspects of women’s empowerment relevant to the outcomes of agricultural development projects and that was more closely linked to theories of agency.

This initial version of pro-WEAI retains many of the properties of the original WEAI as a counting-based index, most importantly, its ability to decompose the overall index value into its sub-indices (3DE and GPI) or its component indicators, as well as by population subgroup. The pro-WEAI responds to the demand of agricultural development projects by including indicators that are relevant to project success, such as indicators of intrinsic agency related to intrahousehold harmony, indicators of intrinsic agency based on well-validated attitudinal questions about IPV against women (Yount et al., 2016, Miedema et al., 2018), and an instrumental agency indicator of women’s freedom of movement also based on survey questions that are validated across groups (Yount et al., 2016) and over time (Cheong et al., 2017). The qualitative work also identified many of these indicators as important to community members. With its three-domain structure, pro-WEAI also has a closer theoretical link to the three domains of empowerment: intrinsic, instrumental, and collective agency.

Because pro-WEAI is based on the same Alkire-Foster methodology as the original WEAI (Alkire et al., 2013), pro-WEAI belongs firmly to the WEAI family of indicators, even if several indicators are new. Pro-WEAI can be decomposed into its component indicators, like the original WEAI. Some versions of WEAI, notably the abbreviated WEAI or A-WEAI, can also be calculated from pro-WEAI because its indicators are nested within pro-WEAI, albeit with different cut-offs and indicator weights. By construction, pro-WEAI will also be comparable across projects because the indicators are defined similarly across all projects. However, as noted previously, because the samples from which pro-WEAI are drawn will not be nationally representative, pro-WEAI diagnostics will not be comparable to those obtained from nationally representative samples.

The process of pro-WEAI development, with sequenced and integrated quantitative and qualitative work, illustrates the value of qualitative work and mixed methods research in general. Although the qualitative work is not a part of the quantitative index, the mixed methods approach followed in the development of pro-WEAI illustrates “pro-WEAI good practice” because qualitative data are valuable for contextualizing the index scores and revealing how project interventions affect women’s empowerment. The qualitative work also showed that despite the variability in local understanding of empowerment, many of the underlying concepts can be mapped to the three domains of empowerment included in pro-WEAI: instrumental, collective, and intrinsic agency.

Pro-WEAI is still in development. Colleagues at Emory University are using IRT methods to assess the measurement properties of a subset of pro-WEAI indicators that were measured in baseline surveys from two GAAP2 projects: the TRAIN project in Bangladesh and the Grameen Foundation project in Burkina Faso (Yount et al., 2019). A health and nutrition module examining instrumental agency related to health and nutrition decisions is being developed and validated with the nutrition-focused projects in the GAAP2 portfolio (Heckert et al., nd); a livestock module is also being developed and tested. Qualitative work is ongoing for some partner projects, and process evaluations are attempting to unpack pathways of impact between project strategies and achieved outcomes. Eventually, this process will result in a standardized pro-WEAI core module and standardized and validated “add-on” modules focusing on specific aspects targeted by projects, like health and nutrition outcomes. These add-on modules would not be used in computing different versions of pro-WEAI but would be used to enhance its usefulness in particular types of projects.

In addition, ongoing work attempts to address several limitations in the pilot survey instrument. For instance, several indicators were developed initially, based on requests from the projects, but ultimately were not included in the index. These indicators include access to information and additional indicators of collective agency. In particular, the survey question, “To what extent are you able to access information that you feel is important for making informed decisions regarding [ACTIVITY]?” included several competing value judgments, which made consistent interpretation difficult. Currently, we are developing an add-on module measuring access to information. We also sought to include more refined indicators of collective agency to balance this domain with intrinsic agency and instrumental agency in pro-WEAI. We experimented with an indicator of whether the respondent felt they had effective voice in groups. Unfortunately, we determined that adding indicators of collective agency beyond the two indicators already included (group membership and membership in influential groups) was not advisable, given that few respondents were group members and all indicators of group membership were drawn from the same survey module. Informed in part by findings from the IRT analysis (Yount et al., 2019), we currently are investigating other approaches to measure collective agency that do not rely explicitly on membership in a group (e.g., engagement in community activities, having shared goals with other women in the same community).^16^

### 5.2 Recommendations on the use of pro-WEAI

Our ongoing work has demonstrated that there is a high potential that pro-WEAI and its component indicators will be sensitive to change over time, especially in terms of measuring project impact. Although the instrument has been developed and piloted in the context of agricultural development projects that aim to empower women, it can be used for other types of projects. Many agricultural development projects are targeted to the whole household, and some of those are in fact gender blind. In such projects, pro-WEAI may be useful for identifying unintended impacts. While we do not recommend pro-WEAI for contexts where agricultural production is not a common livelihood, some of the indicators that are not focused on agricultural production may be useful in non-agricultural settings.

Finally, we emphasize that pro-WEAI is being developed not only to measure empowerment in agricultural development projects, but also to assess projects’ impact on women’s empowerment on other critical economic and social domains, such as savings and borrowing activities, household activities, and more general freedom of movement in public space. The participating projects are conducting endline data collection with the refined survey instrument, with endline results expected in 2020. The pro-WEAI team is awaiting the results of the impact evaluations using the baseline and endline pro-WEAI surveys to be able to say, based on evidence across the 13-project portfolio, what strategies worked to empower women.

## Declaration of Competing Interest

The authors declare that they have no conflicts of interest.

## Appendix A GAAP2 portfolio selection process

To select a portfolio of projects, we issued a call for expressions of interest that resulted in 80 submissions from agricultural development projects in South Asia and Africa South of the Sahara, of which 21 were invited to submit full proposals. Criteria for selection included being gender-aware or gender-sensitive in project design, with a solid M&E framework, and a well-designed impact evaluation plan based on quantitative data and plans (or willingness) to undertake qualitative data collection.

Of the 21 projects, 16 were invited to participate in an inception workshop in which they identified indicators that they thought should be included in pro-WEAI. Despite overall feedback that the WEAI was too long, projects identified several new indicators of empowerment that they wanted included.^17^ Hence, the list of potential indicators, and consequently the baseline data-collection instrument for the pro-WEAI pilot, was even longer than in the WEAI.^18^

Of the 16 projects at the inception workshop, 13 projects were selected for the GAAP2 portfolio. Final selection criteria included ensuring that projects focused on either crops or livestock and had income-oriented or nutrition-oriented objectives (though, in practice, many projects included both crops and livestock, income, and nutrition objectives). The final set of projects is given in Table 1.

These projects provided input in designing the questionnaire, and ultimately, fielded the pilot pro-WEAI survey instrument as part of their impact evaluation efforts. The projects also undertook qualitative work to validate the concepts of empowerment in each context, using protocols that were adopted throughout the portfolio (see Meinzen-Dick et al., 2019).

## Appendix B Computation of the index

Computation of the pro-WEAI follows the methodology of the original WEAI (Alkire et al., 2013). Pro-WEAI is calculated as the weighted mean of two sub-indices: the Three Domains of Empowerment Index (3DE), with a weight of 90 percent, and the Gender Parity Index (GPI), with a weight of 10 percent. The 3DE measures women’s empowerment across three domains: **intrinsic agency** (power within), **instrumental agency** (power to), and **collective agency** (power with). The GPI compares the empowerment scores of the eligible individual and her spouse, or the male respondent, in each household. The choice of weights for the two sub-indices follows the original WEAI, placing greater emphasis on the 3DE while still recognizing the importance of gender equality as an aspect of empowerment. Improvements in either the 3DE or GPI will increase pro-WEAI scores.

### Appendix B.1 Three Domains of Empowerment Index (3DE)

To measure empowerment, we focus on the areas of disempowerment that must be overcome. We start by computing an index of disempowerment, $M_{0}$, using the Alkire-Foster method—an axiomatic and counting-based approach designed originally for measuring multidimensional poverty (Alkire & Foster, 2011). This index captures the percentage of women who are disempowered, as well as the average share of inadequacies that they experience. This index varies between 0, when no one is disempowered, and 1, when everyone is disempowered and inadequate in all indicators. The 3DE is defined as (1 – $M_{0}$). This approach focuses on disempowered women and allows us to identify the key issues that need to be addressed to increase empowerment. We describe below the steps to compute the 3DE using a notation consistent with the $M_{0}$ measurement (Alkire & Foster, 2011).

(i) Identify inadequacies. For each of the 12 indicators described in the previous section, a person is identified as adequate or inadequate. Person i is inadequate in indicator j if his or her level of achievement, $x_{\mathrm{ij}}$, is below the adequacy cut-off $z_{j}$. To each person in each indicator, we assign an inadequacy status $g_{\mathrm{ij}}=1$, if $x_{\mathrm{ij}}<z_{j}$, and $g_{\mathrm{ij}}=0$, otherwise.

(ii) Create the inadequacy score. For each person, the inadequacy score, $c_{i}$, is calculated by summing the inadequacy status of all indicators, each multiplied by their corresponding weight ${(w}_{j})$. More formally, $c_{i}=\sum_{j=1}^{12}w_{j}\times g_{\mathrm{ij}}$. In pro-WEAI, all 12 indicators are equally weighted, and thus $w_{j}=1/12$. The inadequacy score represents the share of indicators in which a person is inadequate.

(iii) Identify the disempowered. To identify who is disempowered, we compare a person’s inadequacy score with the disempowerment cut-off, $k\in \left(0,1\right]$. The disempowerment cut-off is the share of (weighted) inadequacies an individual must have to be considered disempowered*.* Thus, a person is identified as disempowered if $c_{i}>k$, and empowered, otherwise.^19^ In pro-WEAI, k is set at 0.25, and thus a person is identified as disempowered if they are inadequate in at least 4 of the 12 indicators.

(iv) Compute the disempowerment headcount ratio. The disempowerment headcount ratio or the percentage of women who are disempowered, $H_{p}$, is $\frac{q}{n}$, whereq is the number of women identified as disempowered and n is the total number of women.

(v) Compute the intensity of disempowerment. To focus measurement on the situation of the disempowered, we censor the inadequacy scores.^20^ The censored inadequacy score, $c_{i}\left(k\right)$, for individual i is equal to the inadequacy score if the individual is disempowered (i.e., if $c_{i}>k$, then $c_{i}\left(k\right)=c_{i}$). The censored inadequacy score, $c_{i}\left(k\right)$, is equal to zero if the individual is empowered (i.e., if $c_{i}\leq k$, then $c_{i}\left(k\right)=0$). The intensity (or breadth) of disempowerment ($A_{p}$) is the average inadequacy score of disempowered women:

$$A_{p}=\frac{\sum_{i=1}^{n}c_{i}\left(k\right)}{q}.$$

(vi) Compute the index of disempowerment *M*_0_ and the 3DE. With $M_{0}$, the disempowerment headcount ratio is adjusted for the intensity of disempowerment. $M_{0}$ is calculated as the product of the disempowerment headcount ratio and the intensity of disempowerment, $M_{0}=H_{p}\times A_{p}$, or, more simply, as the average censored inadequacy score among women:

$$M_{0}=\frac{1}{n}\sum_{i=1}^{n}c_{i}\left(k\right).$$

The 3DE is easily obtained:

$$3DE=1-M_{0}=1-\left(H_{p}\times A_{p}\right).$$

Although based on *M*_0_, the 3DE also can be expressed equivalently as:

$$3DE=H_{e}+\left(H_{p}\times A_{e}\right),$$

where $H_{e}$ is the empowered headcount ratio, which equals (1– $H_{p}$), and $A_{e}$ is the average adequacy score of disempowered women, which equals (1 – $A_{p}$).

*M*_0_ has two properties that can be useful for understanding disempowerment and analyzing the effects of a project: dimensional breakdown and subgroup decomposition.

a) Dimensional breakdown

*M*_0_ can be decomposed into the contribution of each indicator. This can be useful for diagnostic purposes—understanding which indicators to target to achieve greater increases in empowerment—and reveals broad patterns of how people are disempowered. Continuing our focus on the inadequacies of the disempowered, we begin the decomposition by censoring the inadequacy status for each individual, replacing with zero the inadequacies of the empowered (as above, $g_{\mathrm{ij}}\left(k\right)=g_{\mathrm{ij}}$ if $c_{i}\geq k$ and $g_{\mathrm{ij}}\left(k\right)=0$, otherwise). Then, we compute the censored inadequacy headcount ratios. The censored inadequacy headcount ratio of indicator j, denoted $h_{j}\left(k\right)$, is the proportion of the population that is both disempowered and simultaneously inadequate in that indicator. Formally:

$$h_{j}\left(k\right)=\frac{1}{n}\sum_{i=1}^{n}g_{\mathrm{ij}}\left(k\right).$$

Thus, *M*_0_ can also be written as the weighted sum of the censored headcount ratios:

$$M_{0}=\sum_{j=1}^{d}w_{j}\times h_{j}\left(k\right).$$

The absolute contribution to disempowerment of indicator j is $w_{j}\times h_{j}\left(k\right)$ and the relative contribution is $\frac{w_{j}\times h_{j}\left(k\right)}{M_{0}}$. Whenever the relative contribution to disempowerment of an indicator greatly exceeds its weight, this suggests that the disempowered are disproportionally more inadequate in this indicator compared to other indicators.

## b) Subgroup decomposition

*M*_0_ also can be disaggregated by subgroups, such as treatment arms, depending on sample design and as long as the respective groups are mutually exclusive and exhaustive of the total sample (Alkire et al., 2015).^21^ Disaggregating *M*_0_, and more generally pro-WEAI, by subgroup requires that the underlying data are statistically representative of the subgroup. The subgroup decomposition is calculated as:

$$M_{0}=\sum_{l=1}^{m}\frac{n^{l}}{n}M_{0}^{l},$$

where $M_{0}^{l}$ denotes the *M*_0_ of group $l\in \left[1,m\right]$ and $\frac{n^{l}}{n}$ denotes the population share of that same group. Thus, the relative contribution to disempowerment of group l is $\frac{\frac{n^{l}}{n}\times M_{0}^{l}}{M_{0}}$. As before, whenever the relative contribution to disempowerment of a group greatly exceeds its population share, this suggests that the group may bear a disproportionate share of disempowerment.

### Appendix B.2 Gender Parity Index (GPI)

The GPI focuses on the difference between the inadequacy scores of the eligible woman and her spouse within each household. In contrast to the 3DE, which focuses on women’s inadequacy scores and is based on the full sample of women, the GPI involves the calculation of inadequacy scores for men and women and is based on the sample of dual-adult households (i.e., comprised of at least one woman and one man). Although in most cases the two adults compared will be a woman and her spouse, this is not a requirement.

Similar to the 3DE, the GPI is defined in terms of empowerment. Its construction stems from the identification of households that lack gender parity. The steps to construct the GPI are described below.

(i) Censor the inadequacy scores for gender parity. The inadequacy scores of men or women who are empowered, i.e., whose inadequacy scores are less than or equal to the disempowerment cut-off k, are replaced by the value of k (rather than zero as in the computation of the 3DE). The new censored inadequacy score, denoted as ${c^{\text{'}}}_{i}\left(k\right)$ to differentiate it from the 3DE, is defined as follows: ${c\text{'}}_{i}\left(k\right)=c_{i}$ if $c_{i}>k$, and ${c\text{'}}_{i}\left(k\right)=k$ if $c_{i}\leq k$.

(ii) Identify households lacking gender parity. A household lacks gender parity if the woman is disempowered and her new censored inadequacy score is higher than the new censored inadequacy score of her male counterpart. Formally, household j lacks gender parity if ${{c\text{'}}_{j}\left(k\right)}^{W}>k$ and ${{c^{\text{'}}}_{j}\left(k\right)}^{W}>{{c^{\text{'}}}_{j}\left(k\right)}^{M}$, where ${{c\text{'}}_{j}\left(k\right)}^{W}$ and ${{c\text{'}}_{j}\left(k\right)}^{M}$ are the censored inadequacy scores of the eligible woman and spouse, respectively. Put differently, a household is identified as achieving gender parity if the woman is empowered or, if she is not empowered, her inadequacy score is equal or lower than that of the man in her household.

(iv) Compute the proportion of households lacking gender parity. The proportion of households where women lack gender parity relative to their male counterparts, ${(H}_{\mathrm{GPI}})$ is r/m, where *r* is the number of households classified as lacking gender parity and *m* is the total number of dual-adult households in the sample.

(v) Compute the average empowerment gap. The empowerment gap captures the extent of the disparity between women’s and men’s inadequacy scores in households that lack gender parity. It is calculated as the average relative gap in the censored inadequacy scores between women and men living in households that lack gender parity:

$$I_{\mathrm{GPI}}=\frac{1}{r}\sum_{j=1}^{r}\frac{{{c\text{'}}_{j}\left(k\right)}^{W}-{{c\text{'}}_{j}\left(k\right)}^{M}}{1-{{c\text{'}}_{j}\left(k\right)}^{M}}$$

(vi) Computing the GPI. The GPI combines the two last figures: the percentage of women who lack gender parity and the average empowerment gap:^22^

$$\mathrm{GPI}=1-{(H}_{\mathrm{GPI}}\times I_{\mathrm{GPI}}).$$

Like the 3DE, the GPI is decomposable by subgroups.

## Footnotes

2 Rowlands only refers to “power to” and “power with” as generative, but we take the perspective that “power within” is also a form of generative power.

3 The counting-based approach distinguishes the WEAI from other index-based or scale-based approaches. It enables us to count both disempowered women and the numbers of indicators in which they are disempowered (or inadequate).

4 Best practice requires that modifications of the WEAI should cite which aspects of the WEAI were used, and how it was modified. It is not appropriate to call a modified version “WEAI” unless key elements of the methodology are preserved.

5 Although the WELI evolved separately from the pro-WEAI efforts, the teams from ILRI and IFPRI are now collaborating on a livestock module that can be one of the pro-WEAI add-on modules.

6 See Appendix A for details on the GAAP2 portfolio selection process.

7 A household that includes a male and female adult is called a “dual-adult household” (DHH), and a household with only a female adult is called a “female-adult-only household” (FHH).

8 Ideally, we would have worked only with projects that had not yet started; however, the realities of project implementation schedules and the requirement that all evaluations be completed by the end of the GAAP2 meant that we included some projects that had completed baselines and initiated activities before we developed the questionnaires.

9 For men, rejecting the acceptability of IPV against women is related to intrinsic agency as an indicator of men’s recognition of women’s rights to bodily integrity and freedom from violence, as well as the rejection of “power over” as an aspect of empowerment.

10 WEAI and pro-WEAI share the same underlying methodology, mathematical properties, and structure. However, pro-WEAI is not a mere improvement on the WEAI, rather it is designed for a different purpose, which is to measure empowerment impacts of agricultural development interventions. Aside from measuring new indicators which projects identified as important, pro-WEAI also establishes a higher bar for adequacy to measure the same things as in WEAI.

11 Intuitively, equal weighting by domain makes sense if we think that the three types of agency are equally important. However, given that the number of indicators is not balanced across the three domains, this would mean a heavier weight would be assigned to the collective agency indicators compared to the intrinsic and instrumental agency indicators. Ultimately, in the interest of simplicity and transparency, we opted for equal weighting because there was no a priori rationale for why some indicators would be more important than others. Although weights could be based on local priorities, investigating what those priorities are would be a separate research undertaking; weights that differ depending on location would also not permit comparability across a project portfolio. In the absence of a theoretical rationale for weighting some higher than others, we give all indicators equal weights but perform sensitivity tests on various weighting schemes (see Section 4.1.3, Table 11). Weights also may be derived in the future from our on-going measurement work (Yount et al., 2019).

12 In a previous study in Kenya, for example, Bernier et al. (2015) found that membership in producer organizations increased men’s information about climate-smart agricultural practices, but for women, membership in religious and civic organizations increased awareness of such practices.

13 See Appendix B for details on the calculation of censored and uncensored headcount ratios.

14 See Appendix B for details on how to calculate the contribution of each to disempowerment.

15 Note that nonresponse does not refer to planned missingness or skip patterns, but rather the respondent’s inability or unwillingness to respond.

16 The original WEAI included an indicator related to speaking in public about issues relevant to the respondent or their community. However, in the roll-out of the WEAI in FTF countries, this survey module proved controversial, particularly in contexts with a history of political conflict where speaking in public was a sensitive issue (Malapit et al., 2017) and was ultimately dropped from A-WEAI.
