# Corrigendum to “Development of the project-level Women's Empowerment in Agriculture Index (pro-WEAI)” [World Development 122 (2019) 675–692]

**DOI:** 10.1016/j.worlddev.2020.105135

**Published:** 2020-12

**Authors:** Hazel Malapit, Agnes Quisumbing, Ruth Meinzen-Dick, Greg Seymour, Elena M. Martinez, Jessica Heckert, Deborah Rubin, Ana Vaz, Kathryn M. Yount, Gender Agriculture Assets Project Phase 2 (GAAP2) Study Team

**Affiliations:** a International Food Policy Research Institute, Washington, DC, USA; b Cultural Practice, LLC, USA; c Oxford Poverty and Human Development Initiative, United Kingdom; d Emory University, USA

The authors regret two errors in the original paper. First, there was an error in the calculation of the original results that affects Fig. 4 and Tables 4, 5, 7, 9, 10, and 11 and the text in three instances. (1) On page 682, section 4.1, second paragraph, the GPI should be given as 0.78. (2) On page 686, section 4.1.3.2, first paragraph, the correlation between input in productive decisions and control over use of income should be given as 0.503 and the correlation between group membership and membership in influential groups should be given as 0.764. (3) On page 687, section 4.1.3.3, third paragraph, Spearman’s rho should be given as 0.964 and Kendall’s tau as 0.911. Second, the project sample sizes were incorrectly reported in the original table and figure notes. The correct project sample sizes are as follows: ANGeL (N = 7500), AVC (N = 960), SE LEVER (N = 2705), TRAIN (N = 9735), and WorldVeg (N = 1302). The corrected tables and figures are included below. The results are not qualitatively different from those published in the original paper. The authors would like to apologise for any inconvenience caused.

**Fig. 4:**
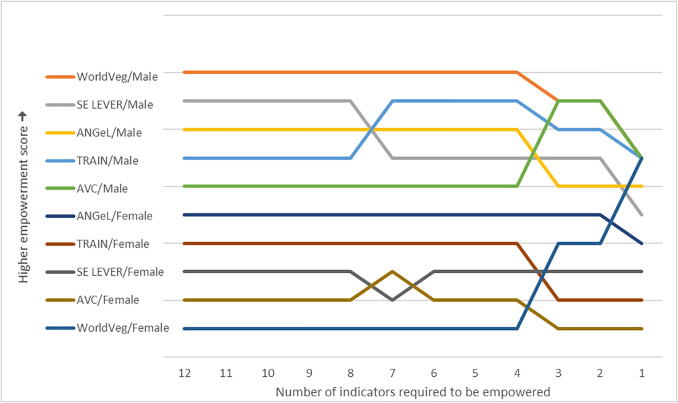
Rank comparison of 3DE scores by project and gender for different empowerment cut-offs. **Source:** Baseline data from ANGeL (N = 7500), AVC (N = 960), SE LEVER (N = 2705), TRAIN (N = 9735), and WorldVeg (N = 1302). **Notes:** 3DE scores ranked from highest to lowest. Spearman’s rho = 1.000; Kendall’s tau b = 1.000. Weighted by inverse project sample size.

**Table 4:** Pro-WEAI results.

Indicator	Women	Men
Number of observations	11513	10689
**3DE score**	**0.57**	**0.76**
Disempowerment score (1—3DE)	0.43	0.24
% achieving empowerment	16%	43%
% not achieving empowerment	84%	57%
Mean adequacy score for not yet empowered	0.49	0.59
Mean disempowerment score (1—adequacy) for not yet empowered	0.51	0.41
Number of dual-adult households	10689	
**Gender Parity Index (GPI)**	**0.78**	
% achieving gender parity	32%	
% not achieving gender parity	68%	
Average empowerment gap	0.33	
**Pro-WEAI score**	**0.59**	

*Source:* Baseline data from ANGeL (N=7500), AVC (N=960), SE LEVER (N=2705), TRAIN (N=9735), and WorldVeg (N=1302).

*Note:* Weighted by inverse project sample size. Respondents with missing indicators are dropped from the sample.

**Table 5:** Headcount ratios and relative contributions of each indicator to disempowerment.

	Uncensored headcount ratio (%)		Censored headcount ratio (%)		Proportional contribution to disempowerment (%)	
Indicator	Men	Women	Men	Women	Men	Women
*Intrinsic agency*						
Autonomy in income	38.7	41.7	26.3	39.2	9.3	7.5
Self-efficacy	36.8	49.3	28.1	46.4	9.9	8.9
Attitudes about intimate partner violence against women	34.6	49.1	25.3	45.5	8.9	8.8
Respect among household members	25.0	38.4	17.7	35.8	6.2	6.9

*Instrumental agency*						
Input in productive decisions	7.4	18.4	6.7	18.2	2.4	3.5
Ownership of land and other assets	1.1	21.6	1.0	20.3	0.3	3.9
Access to and decisions on financial services	24.5	40.4	18.3	38.8	6.5	7.5
Control over use of income	13.4	33.2	11.1	32.4	3.9	6.2
Work balance	33.6	61.5	23.7	55.3	8.4	10.7
Ability to visit important locations	31.7	59.5	25.1	53.1	8.9	10.2

*Collective agency*						
Group membership	63.7	64.7	48.2	61.3	17.0	11.8
Membership in influential groups	71.5	79.1	51.7	72.8	18.2	14.0

*Source:* Baseline data from ANGeL (N=7500), AVC (N=960), SE LEVER (N=2705), TRAIN (N=9735), and WorldVeg (N=1302).

*Notes:* The censored headcount ratio reflects the percent of respondents who are both disempowered and inadequate in the indicator. Uncensored headcount ratio reflects the percent of respondents who are inadequate in the indicator. Weighted by inverse project sample size.

**Table 7:** Pro-WEAI results by age group.

	Age 16–25		Age 26–45		Age 46+	
Indicator	Women	Men	Women	Men	Women	Men
Number of observations	5148	4786	5862	5290	444	399
**3DE score**	**0.58**	**0.76**	**0.63**	**0.77**	**0.58**	**0.74**
Disempowerment score (1 – 3DE)	0.42	0.24	0.37	0.23	0.42	0.26
% achieving empowerment	18%	40%	23%	44%	17%	40%
% not achieving empowerment	82%	60%	77%	56%	83%	60%
Mean 3DE score for not yet empowered	0.49	0.59	0.52	0.6	0.49	0.58
Mean disempowerment score (1 – 3DE)	0.51	0.41	0.48	0.4	0.51	0.42
Number of dual-adult households	4786		5290		399	
**Gender Parity Index (GPI)**	**0.78**		**0.83**		**0.80**	
% achieving gender parity	34%		42%		38%	
% not achieving gender parity	66%		58%		62%	
Average empowerment gap	0.34		0.29		0.33	
**Pro-WEAI score**	**0.60**		**0.65**		**0.60**	

*Source:* Baseline data from ANGeL (N=7500), AVC (N=960), SE LEVER (N=2705), TRAIN (N=9735), and WorldVeg (N=1302).

*Note:* Weighted by inverse project sample size.

**Table 9:** Association (Cramer’s V) between pro-WEAI indicators.

	Autonomy in income	Self-efficacy	Attitudes about intimate partner violence against women	Respect among household members	Input in productive decisions	Ownership of land and other assets
*Intrinsic agency*						
Autonomy in income	1.000					
Self-efficacy	0.031	1.000				
Attitudes about intimate partner violence against women	0.103	0.056	1.000			
Respect among household members	0.055	0.151	0.079	1.000		

*Instrumental agency*						
Input in productive decisions	0.081	0.064	0.046	0.059	1.000	
Ownership of land and other assets	−0.038	0.106	−0.023	0.091	0.138	1.000
Access to and decisions on financial services	0.087	0.060	0.040	0.015	0.114	0.044
Control over use of income	0.086	0.076	0.121	0.063	0.503	0.080
Work balance	−0.040	0.024	0.061	−0.001	0.032	0.031
Ability to visit important locations	−0.075	0.096	−0.025	0.048	0.083	0.188

*Collective agency*						
Group membership	−0.023	0.020	−0.007	−0.051	0.062	0.028
Membership in influential groups	−0.042	0.032	−0.015	−0.041	0.088	0.088

	Access to and decisions on financial services	Control over use of income	Work balance	Ability to visit important locations	Group membership	Membership in influential groups

*Instrumental agency*						
Access to and decisions on financial services	1.000					
Control over use of income	0.139	1.000				
Work balance	0.039	0.096	1.000			
Ability to visit important locations	0.054	0.028	0.038	1.000		

*Collective agency*						
Group membership	0.094	0.032	0.030	0.105	1.000	
Membership in influential groups	0.061	0.056	0.067	0.125	0.764	1.000

*Source:* Baseline data from ANGeL (N=7500), AVC (N=960), SE LEVER (N=2705), TRAIN (N=9735), and WorldVeg (N=1302).

**Table 10:** Redundancy between pro-WEAI indicators.

	Autonomy in income	Self-efficacy	Attitudes about intimate partner violence against women	Respect among household members	Input in productive decisions	Ownership of land and other assets
*Intrinsic agency*						
Autonomy in income	1.000					
Self-efficacy	0.610	1.000				
Attitudes about intimate partner violence against women	0.640	0.603	1.000			
Respect among household members	0.701	0.742	0.712	1.000		

*Instrumental agency*						
Input in productive decisions	0.891	0.888	0.882	0.883	1.000	
Ownership of land and other assets	0.872	0.912	0.876	0.903	0.900	1.000
Access to and decisions on financial services	0.706	0.698	0.689	0.685	0.896	0.893
Control over use of income	0.793	0.791	0.807	0.782	0.964	0.897
Work balance	0.578	0.578	0.608	0.680	0.879	0.892
Ability to visit important locations	0.563	0.611	0.568	0.701	0.895	0.939

*Collective agency*						
Group membership	0.582	0.580	0.575	0.648	0.897	0.895
Membership in influential groups	0.561	0.595	0.566	0.647	0.921	0.932

	Access to and decisions on financial services	Control over use of income	Work balance	Ability to visit important locations	Group membership	Membership in influential groups

*Instrumental agency*						
Access to and decisions on financial services	1.000					
Control over use of income	0.804	1.000				
Work balance	0.690	0.802	1.000			
Ability to visit important locations	0.696	0.774	0.557	1.000		

*Collective agency*						
Group membership	0.732	0.781	0.540	0.608	1.000	
Membership in influential groups	0.723	0.805	0.578	0.648	1.000	1.000

*Source:* Baseline data from ANGeL (N=7500), AVC (N=960), SE LEVER (N=2705), TRAIN (N=9735), and WorldVeg (N=1302).

**Table 11:** Rank of 3DE scores by project and gender for different weighting schemes.

Project/Gender	Equally weighted by indicator	Equally weighted by domain
WorldVeg/Male	1	1
SE LEVER/Male	2	2
ANGeL/Male	3	3
**TRAIN/Male**	**4**	**6**
**AVC/Male**	**5**	**4**
**ANGeL/Female**	**6**	**5**
TRAIN/Female	7	7
SE LEVER/Female	8	8
AVC/Female	9	9
WorldVeg/Female	10	10

*Source:* Baseline data from ANGeL (N=7500), AVC (N=960), SE LEVER (N=2705), TRAIN (N=9735), and WorldVeg (N=1302).

*Notes:* 3DE scores ranked from highest to lowest (1=highest score; 10=lowest score). Spearman’s rho=0.964; Kendall’s tau b=0.911. Groups where ranking differs in bold. Weighted by inverse project sample size.

