# Supplementary material for: Development of the project-level Women’s Empowerment in Agriculture Index (pro-WEAI)
Source: World Dev. 2019 Oct;122:675–92. doi: 10.1016/j.worlddev.2019.06.018 (PMC6694750; doi:10.1016/j.worlddev.2019.06.018)
Supplement: Supplementary data 1 [file mmc1.docx]

# Appendix C: Pro-WEAI survey modules

MODULE G. WOMEN’S EMPOWERMENT IN AGRICULTURE INDEX – Pilot Pro-WEAI Version

| **Note to survey designers:** The information in module G1 can be captured in different ways; however, there must be a way to: (a) identify the proper individual within the household to be asked the survey, (b) link this individual from the module to the household roster, (c) code the outcome of the interview, especially if the individual is not available, to distinguish this from missing data, and (d) record who else in the household was present during the interview. This instrument must be adapted for country context including adding relevant examples and translations into local languages when appropriate.  ***Note to enumerators:*** *This questionnaire should be administered separately to the primary and secondary respondents identified in the household roster of the household level questionnaire. You should complete this coversheet for each individual identified in the “selection section” even if the individual is not available to be interviewed for reporting purposes. For some surveys (such as those focusing on nutrition outcomes), the female respondent may be the beneficiary woman or mother or primary caregiver of the index child (also the respondent for the pro-WEAI nutrition module). Please make sure that she is also the person interviewed for this questionnaire and that the male respondent is her spouse/partner (if applicable).*  Please double-check to ensure:  You have completed the roster section of the household questionnaire to identify the correct primary and/or secondary respondent(s);  You have noted the household ID and individual ID correctly for the person you are about to interview;  You have gained informed consent from the individual in the household questionnaire;  You have sought to interview the individual in private or where other members of the household cannot overhear or contribute answers.  Do not attempt to make responses between the primary and secondary respondents the same—it is okay for them to be different. |
| --- |

**MODULE G1. INDIVIDUAL IDENTIFICATION**

| **G1.01. Household Identification:** | \|  \|  \|  \|  \|  \|  \| \| --- \| --- \| --- \| --- \| --- \| --- \| | **G1.04 TYPE OF HOUSEHOLD** | Male and female adult…………………………………………………………1  Female adult only………………………………………………………………..2 |
| --- | --- | --- | --- | --- | --- | --- | --- | --- | --- |
| **G1.02. Name of respondent currently being interviewed (ID Code from roster in Section B Household Roster):** | \|  \|  \| \| --- \| --- \| | **G1.05. Outcome of interview:**  **CIRCLE ONE** | Completed……………………………………………………………………………1  HOUSEHOLD MEMBER TOO ILL TO RESPOND/COGNITIVELY IMPAIRED…2  RESPONDENT Not at home/temporarily unavailable……………….3  RESPONDENT Not at home/extended absence…………………………4  REFUSED…………………………………………………………………………….…5  Could not locate…………………………………………………………………6 |
| **Surname, OTHER NAME: ____________________________________________________** | |  |  |
| **G1.03. Sex of respondent:** | Male…………………………….1  FEMale…………………………2 | **G1.06. Ability to be interviewed alone:**  **CIRCLE ONE** | Alone…………………………………………………………………………………..1  With adult females present…………………………………………………2  With adult males present…………………………………………………….3  With adults of BOTH sex present………………………………………….4  With children present…………………………………………………………5  With adults OF BOTH sex and children present…………………….6 |

| **HOUSEHOLD IDENTIFICATION (IN DATA FILE, EACH SUB-MODULE (G2-G8) MUST BE LINKED WITH A HH AND RESPONDENT ID)** | **HOUSEHOLD ID** |  |  |  |  |  |  |
| --- | --- | --- | --- | --- | --- | --- | --- |
|  | **RESPONDENT ID** | | | | |  |  |

**MODULE G2: ROLE IN HOUSEHOLD DECISION-MAKING AROUND PRODUCTION AND INCOME**

| Now I’d like to ask you some questions about your participation in certain types of work activities and on making decisions on various aspects of household life. | | Did you [NAME] participate in [ACTIVITY] in the past 12 months (that is, during the last [one/two] cropping seasons), from [PRESENT MONTH] last year to [PRESENT MONTH] this year? | When decisions are made regarding [ACTIVITY], who is it that normally takes the decision?  **ENTER UP TO THREE (3) MEMBER IDs**  **IF RESPONSE IS MEMBER ID (SELF) ONLY** 🡪 ***G2.05***  **OTHER CODES:**  NON-HH MEMBER...….94  NOT APPLICABLE….…98 🡪 ***NEXT ACTIVITY*** | | | How much input did you have in making decisions about [ACTIVITY]?  **USE CODE G2↓** | To what extent do you feel you can participate in decisions regarding [ACTIVITY] if you want(ed) to?  **CIRCLE ONE** | To what extent are you able to access information that you feel is important for making informed decisions regarding [ACTIVITY]?  **CIRCLE ONE** | How much input did you have in decisions about how much of the outputs of [ACTIVITY] to keep for consumption at home rather than selling?  **USE CODE G2↓** | How much input did you have in decisions about how to use income generated from [ACTIVITY]?  **USE CODE G2↓** |
| --- | --- | --- | --- | --- | --- | --- | --- | --- | --- | --- |
| **ACTIVITY** | | **G2.01** | **G2.02** | | | **G2.03** | **G2.04** | **G2.05** | **G2.06** | **G2.07** |
|  |  |  | **ID #1** | **ID #2** | **ID #3** |  |  |  |  |  |
| **A** | Staple grain farming and processing of the harvest: grains that are grown primarily for food consumption (rice, maize, wheat, millet) | Yes…...1  No…….2 🡪 ***activity B*** |  |  |  |  | Not at all……….…1  Small extent……..2  Medium extent…...3  To a high extent...4 | Not at all……….…1  Small extent……..2  Medium extent…...3  To a high extent...4 |  |  |
| **B** | Horticultural (gardens) or high value crop farming and processing of the harvest | Yes…...1  No…….2 🡪 ***activity C*** |  |  |  |  | Not at all……….…1  Small extent……..2  Medium extent…...3  To a high extent...4 | Not at all……….…1  Small extent……..2  Medium extent…...3  To a high extent...4 |  |  |
| **C** | Large livestock raising (cattle, buffaloes) and processing of milk and/or meat | Yes…...1  No…….2 🡪 ***activity D*** |  |  |  |  | Not at all……….…1  Small extent……..2  Medium extent…...3  To a high extent...4 | Not at all……….…1  Small extent……..2  Medium extent…...3  To a high extent...4 |  |  |
| **D** | Small livestock raising (sheep, goats, pigs) and processing of milk and/or meat | Yes…...1  No…….2 🡪 ***activity E*** |  |  |  |  | Not at all……….…1  Small extent……..2  Medium extent…...3  To a high extent...4 | Not at all……….…1  Small extent……..2  Medium extent…...3  To a high extent...4 |  |  |
| **E** | Poultry and other small animals raising (chickens, ducks, turkeys) and processing of eggs and/or meat | Yes…...1  No…….2 🡪 ***activity F*** |  |  |  |  | Not at all……….…1  Small extent……..2  Medium extent…...3  To a high extent...4 | Not at all……….…1  Small extent……..2  Medium extent…...3  To a high extent...4 |  |  |

| **CODE G2** |
| --- |
| LITTLE TO NO INPUT IN DECISIONS 1  Input into some decisions 2  Input into most OR ALL decisions 3  NOT APPICABLE / No decision made 98 |

|  | | Did you [NAME] participate in [ACTIVITY] in the past 12 months (that is, during the last [one/two] cropping seasons), from [PRESENT MONTH] last year to [PRESENT MONTH] this year? | When decisions are made regarding [ACTIVITY], who is it that normally takes the decision?  **ENTER UP TO THREE (3) MEMBER IDs**  **IF RESPONSE IS MEMBER ID (SELF) ONLY** 🡪 ***G2.05***  **OTHER CODES:**  NON-HH MEMBER...….94  NOT APPLICABLE….…98 🡪 ***NEXT ACTIVITY*** | | | | How much input did you have in making decisions about [ACTIVITY]?  **USE CODE G2↓** | To what extent do you feel you can participate in decisions regarding [ACTIVITY] if you want(ed) to?  **CIRCLE ONE** | To what extent are you able to access information that you feel is important for making informed decisions regarding [ACTIVITY]?  **CIRCLE ONE** | How much input did you have in decisions about how much of the outputs of [ACTIVITY] to keep for consumption at home rather than selling?  **USE CODE G2↓** | How much input did you have in decisions about how to use income generated from [ACTIVITY]?  **USE CODE G2↓** |
| --- | --- | --- | --- | --- | --- | --- | --- | --- | --- | --- | --- |
| **ACTIVITY** | | **G2.01** | **G2.02** | | | | **G2.03** | **G2.04** | **G2.05** | **G2.06** | **G2.07** |
|  |  |  | **ID #1** | | **ID #2** | **ID #3** |  |  |  |  |  |
| **F** | Fishpond culture | Yes…...1  No…….2 🡪 ***activity G*** |  | |  |  |  | Not at all……….…1  Small extent……..2  Medium extent…...3  To a high extent...4 | Not at all……….…1  Small extent……..2  Medium extent…...3  To a high extent...4 |  |  |
| **G** | Non-farm economic activities (running a small business, self-employment, buy-and-sell) | Yes…...1  No…….2 🡪 ***activity H*** |  | |  |  |  | Not at all……….…1  Small extent……..2  Medium extent…...3  To a high extent...4 | Not at all……….…1  Small extent……..2  Medium extent…...3  To a high extent...4 |  |  |
| **H** | Wage and salary employment (work that is paid for in cash or in-kind, including both agriculture and other wage work) | Yes…...1  No…….2 🡪 ***activity I*** | |  |  |  |  | Not at all……….…1  Small extent……..2  Medium extent…...3  To a high extent...4 | Not at all……….…1  Small extent……..2  Medium extent…...3  To a high extent...4 |  |  |
| **I** | Large, occasional household purchases (bicycles, land, transport vehicles) |  | |  |  |  |  | Not at all……….…1  Small extent……..2  Medium extent…...3  To a high extent...4 | Not at all……….…1  Small extent……..2  Medium extent…...3  To a high extent...4 |  |  |
| **J** | Routine household purchases (food for daily consumption or other household needs) |  | |  |  |  |  | Not at all……….…1  Small extent……..2  Medium extent…...3  To a high extent...4 | Not at all……….…1  Small extent……..2  Medium extent…...3  To a high extent...4 |  |  |

| **CODE G2** |
| --- |
| LITTLE TO NO INPUT IN DECISIONS 1  Input into some decisions 2  Input into most OR ALL decisions 3  NOT APPLICABLE / NO DECISION MADE 98 |

|  | **HOUSEHOLD ID** |  |  |  |  |  |  |
| --- | --- | --- | --- | --- | --- | --- | --- |
|  | **RESPONDENT ID** | | | | |  |  |

**MODULE G3(A): ACCESS TO PRODUCTIVE CAPITAL**

| Now I’d like to ask you specifically about your household’s land. | | | | |
| --- | --- | --- | --- | --- |
| **QUESTION** | | **RESPONSE** | | |
| **G3.01.** Does anyone in your household currently own or cultivate land? | | YES……..1  NO………2 🡪 ***G3.06, ITEM A*** | | |
| **G3.02.** Who generally makes decisions about what to plant on this land? | **ENTER UP TO THREE (3) MEMBER IDs**  **OTHER CODES:**  NON-HH MEMBER……………………….94  NOT APPLICABLE………………………..98 | **ID #1** | **ID #2** | **ID #3** |
|  |  |  |  |  |
| **G3.03.** Do you [NAME] solely or jointly cultivate any land? | **CIRCLE ONE** | YES, SOLELY 1  YES, JOINTLY 2  YES, SOLELY AND JOINTLY 3  NO 4 | | |
| **G3.04.** Who generally makes decisions about what to plant on the land that you yourself cultivate? | **ENTER UP TO THREE (3) MEMBER IDs**  **OTHER CODES:**  NON-HH MEMBER……………………….94  NOT APPLICABLE………………………..98 | **ID #1** | **ID #2** | **ID #3** |
|  |  |  |  |  |
| **G3.05.** Do you own any of the land owned or cultivated by your household? | **CIRCLE ONE** | YES, SOLELY 1  YES, JOINTLY 2  YES, SOLELY AND JOINTLY 3  NO 4 | | |

| Now I’d like to ask you about a number of items that could be used to generate income. | | Does anyone in your household currently have any [ITEM]? | Do you [NAME] own any [ITEM]?  **CIRCLE ONE** |
| --- | --- | --- | --- |
| **ITEM** | | **G3.06** | **G3.07** |
| **A** | Large livestock (cattle, buffaloes) | YES……..1  NO………2 🡪 ***item B*** | YES, SOLELY 1  YES, JOINTLY 2  YES, SOLELY AND JOINTLY 3  NO 4 |
| **B** | Small livestock (sheep, goats, pigs) | YES……..1  NO………2 🡪 ***item C*** | YES, SOLELY 1  YES, JOINTLY 2  YES, SOLELY AND JOINTLY 3  NO 4 |
| **C** | Poultry and other small animals (chickens, ducks, turkeys) | YES……..1  NO………2 🡪 ***item D*** | YES, SOLELY 1  YES, JOINTLY 2  YES, SOLELY AND JOINTLY 3  NO 4 |
| **D** | Fish pond or fishing equipment | YES……..1  NO………2 🡪 ***item E*** | YES, SOLELY 1  YES, JOINTLY 2  YES, SOLELY AND JOINTLY 3  NO 4 |
| **E** | Non-mechanized farm equipment (hand tools, animal-drawn plough) | YES……..1  NO………2 🡪 ***item F*** | YES, SOLELY 1  YES, JOINTLY 2  YES, SOLELY AND JOINTLY 3  NO 4 |
| **F** | Mechanized farm equipment (tractor-plough, power tiller, treadle pump) | YES……..1  NO………2 🡪 ***item G*** | YES, SOLELY 1  YES, JOINTLY 2  YES, SOLELY AND JOINTLY 3  NO 4 |
| **G** | Non-farm business equipment (solar panels used for recharging, sewing machine, brewing equipment, fryers) | YES……..1  NO………2 🡪 ***item H*** | YES, SOLELY 1  YES, JOINTLY 2  YES, SOLELY AND JOINTLY 3  NO 4 |
| **H** | House or building | YES……..1  NO………2 🡪 ***item I*** | YES, SOLELY 1  YES, JOINTLY 2  YES, SOLELY AND JOINTLY 3  NO 4 |
| **I** | Large consumer durables (refrigerator, TV, sofa) | YES……..1  NO………2 🡪 ***item J*** | YES, SOLELY 1  YES, JOINTLY 2  YES, SOLELY AND JOINTLY 3  NO 4 |

|  | | Does anyone in your household currently own any [ITEM]? | Do you [NAME] own any [ITEM]?  **CIRCLE ONE** |
| --- | --- | --- | --- |
| **ITEM** | | **G3.06** | **G3.07** |
| **J** | Small consumer durables (radio, cookware) | YES……..1  NO………2 🡪 ***item K*** | YES, SOLELY 1  YES, JOINTLY 2  YES, SOLELY AND JOINTLY 3  NO 4 |
| **K** | Cell phone | YES……..1  NO………2 🡪 ***item L*** | YES, SOLELY 1  YES, JOINTLY 2  YES, SOLELY AND JOINTLY 3  NO 4 |
| **L** | Other land not used for agricultural purposes (pieces/plots, residential or commercial land) | YES……..1  NO………2 🡪 ***item M*** | YES, SOLELY 1  YES, JOINTLY 2  YES, SOLELY AND JOINTLY 3  NO 4 |
| **M** | Means of transportation (bicycle, motorcycle, car) | YES……..1  NO………2 🡪 ***MODULE G3(B)*** | YES, SOLELY 1  YES, JOINTLY 2  YES, SOLELY AND JOINTLY 3  NO 4 |

MODULE G3(B): ACCESS TO FINANCIAL SERVICES

| Next I’d like to ask about your household’s experience with borrowing money or other items (in-kind) in the past 12 months. | | Would you or anyone in your household be able to take a loan or borrow cash/in-kind from [SOURCE] if you wanted to? | Has anyone in your household taken any loans or borrowed cash/in-kind from [SOURCE] in the past 12 months?  **CIRCLE ONE** | Who made the decision to borrow from [SOURCE] most of the time?  **ENTER UP TO THREE (3) MEMBER IDs**  **OTHER CODES:**  NON-HH MEMBER...….94  NOT APPLICABLE….…98 | | | Who makes the decision about what to do with the money or item borrowed from [SOURCE] most of the time?  **ENTER UP TO THREE (3) MEMBER IDs**  **OTHER CODES:**  NON-HH MEMBER...….94  NOT APPLICABLE….…98 | | | Who is responsible for repaying the money or item borrowed from [SOURCE]?  **ENTER UP TO THREE (3) MEMBER IDs**  **OTHER CODES:**  NON-HH MEMBER...….94  NOT APPLICABLE….…98 | | |
| --- | --- | --- | --- | --- | --- | --- | --- | --- | --- | --- | --- | --- |
| **Lending sources** | | **G3.08** | **G3.09** | **G3.10** | | | **G3.11** | | | **G3.12** | | |
|  |  |  |  | **ID #1** | **ID #2** | **ID #3** | **ID #1** | **ID #2** | **ID #3** | **ID #1** | **ID #2** | **ID #3** |
| **A** | Non-governmental organization (NGO) | YES...…….1  NO………..2 🡪 ***SOURCE B***  MAYBE.….3 | YES, CASH 1  YES, IN-KIND 2  YES, CASH AND IN-KIND 3  NO 4 ***SOURCE B***  DON’T KNOW 97 |  |  |  |  |  |  |  |  |  |
| **B** | Formal lender (bank/financial institution) | YES...…….1  NO………..2 🡪 ***SOURCE C***  MAYBE.….3 | YES, CASH 1  YES, IN-KIND 2  YES, CASH AND IN-KIND 3  NO 4 ***SOURCE C*** DON’T KNOW 97 |  |  |  |  |  |  |  |  |  |
| **C** | Informal lender | YES...…….1  NO………..2 🡪 ***SOURCE D***  MAYBE.….3 | YES, CASH 1  YES, IN-KIND 2  YES, CASH AND IN-KIND 3  NO 4 ***SOURCE D*** DON’T KNOW 97 |  |  |  |  |  |  |  |  |  |
| **D** | Friends or relatives | YES...…….1  NO………..2 🡪 ***SOURCE E***  MAYBE.….3 | YES, CASH 1  YES, IN-KIND 2  YES, CASH AND IN-KIND 3  NO 4 ***SOURCE E*** DON’T KNOW 97 |  |  |  |  |  |  |  |  |  |
| **E** | Group based micro-finance or lending including VSLAs / SACCOs | YES...…….1  NO………..2 🡪 ***SOURCE F***  MAYBE.….3 | YES, CASH 1  YES, IN-KIND 2  YES, CASH AND IN-KIND 3  NO 4 ***SOURCE F*** DON’T KNOW 97 |  |  |  |  |  |  |  |  |  |
| **F** | Informal credit / savings groups (.e.g., merry-go-rounds, tontines, funeral societies, etc.) | YES...…….1  NO………..2 🡪 ***G3.13***  MAYBE.….3 | YES, CASH 1  YES, IN-KIND 2  YES, CASH AND IN-KIND 3  NO 4 ***G3.13***  DON’T KNOW 97 |  |  |  |  |  |  |  |  |  |

| **G3.13** | An account can be used to save money, to make or receive payments, or to receive wages or financial help. Do you, either by yourself or together with someone else, currently have an account at any of the following places: a bank or other formal institution (e.g., post office)? | YES 1  NO 2  DON’T KNOW 97 |
| --- | --- | --- |

|  | **HOUSEHOLD ID** |  |  |  |  |  |  |
| --- | --- | --- | --- | --- | --- | --- | --- |
|  | **RESPONDENT ID** | | | | |  |  |

**MODULE G4: TIME ALLOCATION**

**G4.01:** Please record a log of the activities for the individual in the last complete 24 hours (starting yesterday morning at 4 am, finishing 3:59 am of the current day). The time intervals are marked in 15 min intervals. mark one activity for each time period by ENTERING THE CORRESPONDING ACTIVITY CODE in the box.

**G4.02:** CHECK THE BOX BELOW IF THE RESPONDENT WAS CARING FOR CHILDREN WHILE PERFORMING EACH ACTIVITY.

| Now I’d like to ask you about how you spent your time during the past 24 hours. We’ll begin from yesterday morning, and continue through to this morning. This will be a detailed accounting. I’m interested in everything you did (i.e. resting, eating, personal care, work inside and outside the home, caring for children, cooking, shopping, socializing, etc.), even if it didn’t take you much time. I’m particularly interested in agricultural activities such as farming, gardening, and livestock raising whether in the field or on the homestead. I’m also interested in how much time you spent caring for children, especially if it happened while you did some other activity (e.g., collecting water while carrying a child or cooking while watching after a sleeping child). | | | | | | | | | | | | | | | | | | | | | | | | | | | | | | | | | | | | | | | | | | | | | | | | | | | | | | | | | | | | | | | | | | | | | | | | | | | | | | | | | | | | | |
| --- | --- | --- | --- | --- | --- | --- | --- | --- | --- | --- | --- | --- | --- | --- | --- | --- | --- | --- | --- | --- | --- | --- | --- | --- | --- | --- | --- | --- | --- | --- | --- | --- | --- | --- | --- | --- | --- | --- | --- | --- | --- | --- | --- | --- | --- | --- | --- | --- | --- | --- | --- | --- | --- | --- | --- | --- | --- | --- | --- | --- | --- | --- | --- | --- | --- | --- | --- | --- | --- | --- | --- | --- | --- | --- | --- | --- | --- | --- | --- | --- | --- | --- | --- | --- | --- |
|  | | **Night** | | | | | | | | | | | | | | | | | | | **Morning** | | | | | | | | **Day** | | | | | | | | | | | | | | | | | | | | | | | | | | | | | | | | | | | | | | | | | | | | | | | | | | | | | | | | |
|  |  | 4:00 | | | | | | | | | | 5:00 | | | | | | | | | 6:00 | | | | | | | | 7:00 | | | | | | | | | 8:00 | | | | | | | 9:00 | | | | | | | | | | 10:00 | | | | | | | 11:00 | | | | | 12:00 | | | | | 13:00 | | | | | 14:00 | | | | | 15:00 | | | |
| **G4.01** Activity **(WRITE ACTIVITY CODE)** | |  | |  | |  | |  | | | |  |  | |  | |  | | | |  |  |  | |  | | | |  | |  | |  | |  | | |  | |  | |  |  | |  | |  | |  | |  | | |  | | |  | |  |  | |  |  |  |  | |  |  |  |  | |  |  |  |  | |  |  |  |  | |  |  |  |  |
| **G4.02** Did you also care for children?  YES..…CHECK BOX  NO…LEAVE BLANK | YES CHECK BOX  NO LEAVE BLANK | □ | | □ | | □ | | □ | | | | □ | □ | | □ | | □ | | | | □ | □ | □ | | □ | | | | □ | | □ | | □ | | □ | | | □ | | □ | | □ | □ | | □ | | □ | | □ | | □ | | | □ | | | □ | | □ | □ | | □ | □ | □ | □ | | □ | □ | □ | □ | | □ | □ | □ | □ | | □ | □ | □ | □ | | □ | □ | □ | □ |
|  | | **Day** | | | | | | | | | | | | | | | | | **Evening** | | | | | | | | **Night** | | | | | | | | | | | | | | | | | | | | | | | | | | | | | | | | | | | | | | | | | | | | | | | | | | | | | | | | | | |
|  |  | 16:00 | | | | | | | | 17:00 | | | | | | | | | 18:00 | | | | | | | | 19:00 | | | | | | | | | 20:00 | | | | | | | | 21:00 | | | | | | | | | 22:00 | | | | | | | | 23:00 | | | | | 24:00 | | | | | 1:00 | | | | | 2:00 | | | | | 3:00 | | | | |
| **G4.01** Activity **(WRITE ACTIVITY CODE)** | | |  | |  | |  | |  | |  | | |  | |  | |  | |  | |  | |  | |  | |  | |  | |  | |  | | |  | |  | |  | |  | | |  | |  | |  | |  | | |  |  | |  | |  | |  |  |  |  | |  |  |  |  | |  |  |  |  | |  |  |  |  | |  |  |  |  |
| **G4.02** Did you also care for children? | YES CHECK BOX  NO LEAVE BLANK | | □ | | □ | | □ | | □ | | □ | | | □ | | □ | | □ | | □ | | □ | | □ | | □ | | □ | | □ | | □ | | □ | | | □ | | □ | | □ | | □ | | | □ | | □ | | □ | | □ | | | □ | □ | | □ | | □ | | □ | □ | □ | □ | | □ | □ | □ | □ | | □ | □ | □ | □ | | □ | □ | □ | □ | | □ | □ | □ | □ |

| **ACTIVITY CODES FOR G4.01** | | | |
| --- | --- | --- | --- |
| A………………Sleeping and resting  B.......................Eating and drinking  C……………...………Personal care  D………….School (incl. homework)  E……………..…Work as employed  F…………….…Own business work  G……………...Staple grain farming | H…………Horticultural (gardens) or high value crop farming  I………………..…..Large livestock raising (cattle, buffaloes)  J……………...….Small livestock raising (sheep, goats, pigs)  K...............................Poultry and other small animals raising  (chickens, ducks, turkeys)  L…………………………………………….…Fishpond culture  M……………………….Commuting (to/from work or school) | N………..Shopping / getting service (incl. health services)  O………………………..…..Weaving / sewing / textile care  P………………………………………………….…..Cooking  Q…..………..Domestic work (incl. fetching water and fuel)  R…………………………………………..Caring for children  S………………………..…..Caring for adults (sick, elderly)  T……………………..…..Traveling (not for work or school) | U………………………...Exercising  V……Social activities and hobbies  W…….…………Religious activities  X………………..….Other (specify) |

| **G4.03.** In the last 24 hours did you work (at home or outside of the home including chores or other domestic activities) less than usual, about the same as usual, or more than usual? | **FOR FEMALES ONLY:**  **DOES RESPONDENT HAVE A CHILD UNDER 5 YEARS OLD?**    YES...…….1 🡪 G4.04  NO………..2 🡪 MODULE G5 | **G4.04.** If you wanted to do something (livelihood-related, training-related, self-care) and could not take your child with you, is there someone who could care for your child in your absence?  YES...…….1 🡪 G4.05  NO………..2 🡪 MODULE G5 | **G4.05.** Who?  **ENTER UP TO THREE (3) MEMBER IDs**  **OTHER CODES:**  NON-HH MEMBER...….94  NOT APPLICABLE….…98 | **ID #1** | **ID #2** | **ID #3** |
| --- | --- | --- | --- | --- | --- | --- |
| LESS THAN USUAL...........................…….1  ABOUT THE SAME AS USUAL…………...2  MORE THAN USUAL……………………….3  **IF RESPONDENT IS MALE 🡪 MODULE G5** |  |  |  |  |  |  |

|  | **HOUSEHOLD ID** |  |  |  |  |  |  |
| --- | --- | --- | --- | --- | --- | --- | --- |
|  | **RESPONDENT ID** | | | | |  |  |

**MODULE G5: GROUP MEMBERSHIP**

| Now I’m going to ask you about groups in the community. These can be either formal or informal and customary groups. | | Is there a [GROUP] in your community? | | | Is this group composed of all male or female or mixed-sex members? | Are you an active member of this [GROUP]? | To what extent do you feel like you can influence decisions in this [GROUP]? | | To what extent does this [GROUP] influence life in the community beyond the group activities? | | |  |  |  |  |  |  |
| --- | --- | --- | --- | --- | --- | --- | --- | --- | --- | --- | --- | --- | --- | --- | --- | --- | --- |
| **Group Categories** | | **G5.01** | | | **G5.02** | **G5.03** | **G5.04** | | **G5.05** | | |  |  |  |  |  |  |
| **A** | Agricultural / livestock / fisheries producer’s group (including marketing groups) | YES 1  NO 2  DON’T KNOW 97 |  | ***GROUP B*** | ALL MALE……………………..1  ALL FEMALE………….……...2  MIXED SEX……………..…….3  DON’T KNOW……………….97 | YES……1  NO..……2 🡪 ***GROUP B*** | NOT AT ALL…………………...1  SMALL EXTENT………………2  MEDIUM EXTENT…………….3  HIGH EXTENT………………...4 | | NOT AT ALL…………………...1  SMALL EXTENT………………2  MEDIUM EXTENT…………….3  HIGH EXTENT………………...4 | | |  |  |  |  |  |  |
| **B** | Water users’ group | YES 1  NO 2  DON’T KNOW 97 |  | ***GROUP C*** | ALL MALE……………………..1  ALL FEMALE………….……...2  MIXED SEX……………..…….3  DON’T KNOW……………….97 | YES……1  NO..……2 🡪 ***GROUP C*** | NOT AT ALL…………………...1  SMALL EXTENT………………2  MEDIUM EXTENT…………….3  HIGH EXTENT………………...4 | | NOT AT ALL…………………...1  SMALL EXTENT………………2  MEDIUM EXTENT…………….3  HIGH EXTENT………………...4 | | |  |  |  |  |  |  |
| **C** | Forest users’ group | YES 1  NO 2  DON’T KNOW 97 |  | ***GROUP D*** | ALL MALE……………………..1  ALL FEMALE………….……...2  MIXED SEX……………..…….3  DON’T KNOW……………….97 | YES……1  NO..……2 🡪 ***GROUP D*** | NOT AT ALL…………………...1  SMALL EXTENT………………2  MEDIUM EXTENT…………….3  HIGH EXTENT………………...4 | | NOT AT ALL…………………...1  SMALL EXTENT………………2  MEDIUM EXTENT…………….3  HIGH EXTENT………………...4 | | |  |  |  |  |  |  |
| **D** | Credit or microfinance group (including SACCOs / merry-go-rounds / VSLAs) | YES 1  NO 2  DON’T KNOW 97 |  | ***GROUP E*** | ALL MALE……………………..1  ALL FEMALE………….……...2  MIXED SEX……………..…….3  DON’T KNOW……………….97 | YES……1  NO..……2 🡪 ***GROUP E*** | NOT AT ALL…………………...1  SMALL EXTENT………………2  MEDIUM EXTENT…………….3  HIGH EXTENT………………...4 | | NOT AT ALL…………………...1  SMALL EXTENT………………2  MEDIUM EXTENT…………….3  HIGH EXTENT………………...4 | | |  |  |  |  |  |  |
| **E** | Mutual help or insurance group (including burial societies) | YES 1  NO 2  DON’T KNOW 97 |  | ***GROUP F*** | ALL MALE……………………..1  ALL FEMALE………….……...2  MIXED SEX……………..…….3  DON’T KNOW……………….97 | YES……1  NO..……2 🡪 ***GROUP F*** | NOT AT ALL…………………...1  SMALL EXTENT………………2  MEDIUM EXTENT…………….3  HIGH EXTENT………………...4 | | NOT AT ALL…………………...1  SMALL EXTENT………………2  MEDIUM EXTENT…………….3  HIGH EXTENT………………...4 | | |  |  |  |  |  |  |
| **F** | Trade and business association group | YES 1  NO 2  DON’T KNOW 97 |  | ***GROUP G*** | ALL MALE……………………..1  ALL FEMALE………….……...2  MIXED SEX……………..…….3  DON’T KNOW……………….97 | YES……1  NO..……2 🡪 ***GROUP G*** | NOT AT ALL…………………...1  SMALL EXTENT………………2  MEDIUM EXTENT…………….3  HIGH EXTENT………………...4 | | NOT AT ALL…………………...1  SMALL EXTENT………………2  MEDIUM EXTENT…………….3  HIGH EXTENT………………...4 | | |  |  |  |  |  |  |
| **G** | Civic group (improving community) or charitable group (helping others) | YES 1  NO 2  DON’T KNOW 97 |  | ***GROUP H*** | ALL MALE……………………..1  ALL FEMALE………….……...2  MIXED SEX……………..…….3  DON’T KNOW……………….97 | YES……1  NO..……2 🡪 ***GROUP H*** | NOT AT ALL…………………...1  SMALL EXTENT………………2  MEDIUM EXTENT…………….3  HIGH EXTENT………………...4 | | NOT AT ALL…………………...1  SMALL EXTENT………………2  MEDIUM EXTENT…………….3  HIGH EXTENT………………...4 | | |  |  |  |  |  |  |
| **H** | Religious group | YES 1  NO 2  DON’T KNOW 97 |  | ***GROUP I*** | ALL MALE……………………..1  ALL FEMALE………….……...2  MIXED SEX……………..…….3  DON’T KNOW……………….97 | YES……1  NO..……2 🡪 ***GROUP I*** | NOT AT ALL…………………...1  SMALL EXTENT………………2  MEDIUM EXTENT…………….3  HIGH EXTENT………………...4 | | NOT AT ALL…………………...1  SMALL EXTENT………………2  MEDIUM EXTENT…………….3  HIGH EXTENT………………...4 | | |  |  |  |  |  |  |
| **I** | Other (specify): _______________________ | YES 1  NO 2  DON’T KNOW 97 |  | ***MODULE G6*** | ALL MALE……………………..1  ALL FEMALE………….……...2  MIXED SEX……………..…….3  DON’T KNOW……………….97 | YES……1  NO..……2 🡪 ***MODULE G6*** | NOT AT ALL…………………...1  SMALL EXTENT………………2  MEDIUM EXTENT…………….3  HIGH EXTENT………………...4 | | NOT AT ALL…………………...1  SMALL EXTENT………………2  MEDIUM EXTENT…………….3  HIGH EXTENT………………...4 | | |  |  |  |  |  |  |
|  | | | | | | | | | **HOUSEHOLD ID** | |  |  | |  |  |  |  |
|  |  |  |  |  |  |  |  |  | **RESPONDENT ID** | | | | | | |  |  |

**MODULE G6. PHYSICAL MOBILITY**

| **QUESTION** | **RESPONSE**  **FOR G6.01 - G6.06: USE CODE G6↓** |
| --- | --- |
| **G6.01** How often do you visit an urban center? |  |
| **G6.02** How often do you go to the market / haat / bazaar? |  |
| **G6.03** How often do you go to visit family or relatives? |  |
| **G6.04** How often do you go to visit a friend / neighbor’s house? |  |
| **G6.05** How often do you go to the hospital / clinic / doctor (seek health service)? |  |
| **G6.06** How often do you go to a public village gathering / community meeting / training for NGO or programs? |  |
| **G6.07.** In the last 12 months, how many times have you been away from home for one or more nights (in other words, sleeping somewhere else for the night)? |  |
| **G6.08.** In the last 12 months, have you been away from home for more than one month at a time? | YES…………………………………………………1  NO…………………………………………………..2  ***IF RESPONDENT IS MALE*** 🡪***MODULE G7*** |

| **CODE G6** |
| --- |
| EVERYDAY 1  EVERY WEEK AT LEAST ONCE 2  EVERY 2 WEEKS AT LEAST ONCE 3  EVERY MONTH AT LEAST ONCE 4  LESS THAN ONCE A MONTH 5  NEVER 6 |

**REMAINDER OF MODULE (G6.09-G6.08) SHOULD ONLY BE ASKED IF RESPONDENT IS FEMALE**

| Now I’d like to ask you some questions about different places you might visit. | | Who usually decides whether you can go to [PLACE]?  **ENTER UP TO THREE (3) MEMBER IDs**  **IF RESPONSE IS MEMBER ID (SELF) ONLY** 🡪 **NEXT PLACE**  **OTHER CODES:**  NON-HH MEMBER...….94  NOT APPLICABLE….…98 | | | Does your husband/partner or other household member object to you going alone to [PLACE]? | Under what circumstances would this person NOT object to your going to [PLACE] alone?  **CIRCLE ALL APPLICABLE** | Do these objections prevent you from going alone to [PLACE]? |
| --- | --- | --- | --- | --- | --- | --- | --- |
| **PLACE** | | **G6.09** | | | **G6.10** | **G6.11** | **G6.12** |
|  |  | **ID #1** | **ID #2** | **ID #3** |  |  |  |
| **A** | Urban center |  |  |  | YES……1  NO..……2 🡪 ***PLACE B*** | IF I HAVE COMPANY (RELATIVES, CHILDREN)………………………..….1  IF I CAN ARRANGE MY OWN EXPENSES (FOR TRANSPORT)………....2  IF I FOLLOW PURDAH / DRESS ACCEPTABLY…………………………...3  OTHER (SPECIFY)………………………………………………………………4  UNDER NO CIRCUMSTANCES WOULD I BE ALLOWED TO GO………..5 🡪 ***PLACE B*** | YES……1  NO..……2 |
| **B** | Market / haat / bazaar |  |  |  | YES……1  NO..……2 🡪 ***PLACE C*** | IF I HAVE COMPANY (RELATIVES, CHILDREN)………………………..….1  IF I CAN ARRANGE MY OWN EXPENSES (FOR TRANSPORT)………....2  IF I FOLLOW PURDAH / DRESS ACCEPTABLY…………………………...3  OTHER (SPECIFY)………………………………………………………………4  UNDER NO CIRCUMSTANCES WOULD I BE ALLOWED TO GO………..5 🡪 ***PLACE C*** | YES……1  NO..……2 |
| **C** | Visit family or relatives |  |  |  | YES……1  NO..……2 🡪 ***PLACE D*** | IF I HAVE COMPANY (RELATIVES, CHILDREN)………………………..….1  IF I CAN ARRANGE MY OWN EXPENSES (FOR TRANSPORT)………....2  IF I FOLLOW PURDAH / DRESS ACCEPTABLY…………………………...3  OTHER (SPECIFY)………………………………………………………………4  UNDER NO CIRCUMSTANCES WOULD I BE ALLOWED TO GO………..5 🡪 ***PLACE D*** | YES……1  NO..……2 |
| **D** | Visit a friend / neighbor’s house |  |  |  | YES……1  NO..……2 🡪 ***PLACE E*** | IF I HAVE COMPANY (RELATIVES, CHILDREN)………………………..….1  IF I CAN ARRANGE MY OWN EXPENSES (FOR TRANSPORT)………....2  IF I FOLLOW PURDAH / DRESS ACCEPTABLY…………………………...3  OTHER (SPECIFY)………………………………………………………………4  UNDER NO CIRCUMSTANCES WOULD I BE ALLOWED TO GO………..5 🡪 ***PLACE E*** | YES……1  NO..……2 |
| **E** | Hospital / clinic / doctor (seek health service) |  |  |  | YES……1  NO..……2 🡪 ***PLACE F*** | IF I HAVE COMPANY (RELATIVES, CHILDREN)………………………..….1  IF I CAN ARRANGE MY OWN EXPENSES (FOR TRANSPORT)………....2  IF I FOLLOW PURDAH / DRESS ACCEPTABLY…………………………...3  OTHER (SPECIFY)………………………………………………………………4  UNDER NO CIRCUMSTANCES WOULD I BE ALLOWED TO GO………..5 🡪 ***PLACE F*** | YES……1  NO..……2 |

|  | | Who usually decides whether you can go to [PLACE]?  **ENTER UP TO THREE (3) MEMBER IDs**  **IF RESPONSE IS MEMBER ID (SELF) ONLY** 🡪 **NEXT PLACE**  **OTHER CODES:**  NON-HH MEMBER...….94  NOT APPLICABLE….…98 | | | Does your husband/partner or other household member object to you going alone to [PLACE]? | Under what circumstances would this person NOT object to your going to [PLACE] alone?  **CIRCLE ALL APPLICABLE** | Do these objections prevent you from going alone to [PLACE]? |
| --- | --- | --- | --- | --- | --- | --- | --- |
| **PLACE** | | **G6.09** | | | **G6.10** | **G6.11** | **G6.12** |
|  |  | **ID #1** | **ID #2** | **ID #3** |  |  |  |
| **F** | Temple / church / mosque |  |  |  | YES……1  NO..……2 🡪 ***PLACE G*** | IF I HAVE COMPANY (RELATIVES, CHILDREN)………………………..….1  IF I CAN ARRANGE MY OWN EXPENSES (FOR TRANSPORT)………....2  IF I FOLLOW PURDAH / DRESS ACCEPTABLY…………………………...3  OTHER (SPECIFY)………………………………………………………………4  UNDER NO CIRCUMSTANCES WOULD I BE ALLOWED TO GO………..5 🡪 ***PLACE G*** | YES……1  NO..……2 |
| **G** | Public village gathering or community meeting |  |  |  | YES……1  NO..……2 🡪 ***PLACE H*** | IF I HAVE COMPANY (RELATIVES, CHILDREN)………………………..….1  IF I CAN ARRANGE MY OWN EXPENSES (FOR TRANSPORT)………....2  IF I FOLLOW PURDAH / DRESS ACCEPTABLY…………………………...3  OTHER (SPECIFY)………………………………………………………………4  UNDER NO CIRCUMSTANCES WOULD I BE ALLOWED TO GO………..5 🡪 ***PLACE H*** | YES……1  NO..……2 |
| **H** | Training for NGO / programs |  |  |  | YES……1  NO..……2 🡪 ***PLACE I*** | IF I HAVE COMPANY (RELATIVES, CHILDREN)………………………..….1  IF I CAN ARRANGE MY OWN EXPENSES (FOR TRANSPORT)………....2  IF I FOLLOW PURDAH / DRESS ACCEPTABLY…………………………...3  OTHER (SPECIFY)………………………………………………………………4  UNDER NO CIRCUMSTANCES WOULD I BE ALLOWED TO GO………..5 🡪 ***PLACE I*** | YES……1  NO..……2 |
| **I** | Outside your community or village |  |  |  | YES……1  NO..……2 🡪 ***MODULE G7*** | IF I HAVE COMPANY (RELATIVES, CHILDREN)………………………..….1  IF I CAN ARRANGE MY OWN EXPENSES (FOR TRANSPORT)………....2  IF I FOLLOW PURDAH / DRESS ACCEPTABLY…………………………...3  OTHER (SPECIFY)………………………………………………………………4  UNDER NO CIRCUMSTANCES WOULD I BE ALLOWED TO GO………..5 🡪 ***MODULE G7*** | YES……1  NO..……2 |

|  | **HOUSEHOLD ID** |  |  |  |  |  |  |
| --- | --- | --- | --- | --- | --- | --- | --- |
|  | **RESPONDENT ID** | | | | |  |  |

**MODULE G7: INTRAHOUSEHOLD RELATIONSHIPS**

| Now I’d like to ask you some questions about how you feel about some of other people in your household or family group and how you think they feel about you.  **ENTER MEMBER ID FOR EACH RELATION**  **OTHER CODES:**  NON-HH MEMBER...….94 | | | Do you [NAME] respect your [RELATION]? | Does your [RELATION] respect you? | Do you trust your [RELATION] to do things that are in your best interest? | When you disagree with your [RELATION], do you feel comfortable telling him/her that you disagree? | **IS [RELATION] THE OTHER RESPONDENT WITHIN THIS HOUSEHOLD?** | Is there a co-wife within your household? |
| --- | --- | --- | --- | --- | --- | --- | --- | --- |
| **RELATION** | | | **G7.02** | **G7.03** | **G7.04** | **G7.05** | **G7.06** | **G7.07** |
| **A** | Husband / wife | **ID #** | MOST OF THE TIME...........1  SOMETIMES………………..2  RARELY……………………..3  NEVER………………………4 | MOST OF THE TIME...........1  SOMETIMES………………..2  RARELY……………………..3  NEVER………………………4 | MOST OF THE TIME...........1  SOMETIMES………………..2  RARELY……………………..3  NEVER………………………4 | MOST OF THE TIME...........1  SOMETIMES………………..2  RARELY……………………..3  NEVER………………………4 | YES……1 🡪 ***RELATION C***  NO..……2 |  |
|  |  |  |  |  |  |  |  |  |
| **B** | Other respondent within the household | **ID #** | MOST OF THE TIME...........1  SOMETIMES………………..2  RARELY……………………..3  NEVER………………………4 | MOST OF THE TIME...........1  SOMETIMES………………..2  RARELY……………………..3  NEVER………………………4 | MOST OF THE TIME...........1  SOMETIMES………………..2  RARELY……………………..3  NEVER………………………4 | MOST OF THE TIME...........1  SOMETIMES………………..2  RARELY……………………..3  NEVER………………………4 |  |  |
|  |  |  |  |  |  |  |  |  |
| **C** | **IF RESPONDENT IS MALE:**  Father (or adapt this category to capture other important relationship)    **IF RESPONDENT IS FEMALE:** Mother-in-law | **ID #** | MOST OF THE TIME...........1  SOMETIMES………………..2  RARELY……………………..3  NEVER………………………4 | MOST OF THE TIME...........1  SOMETIMES………………..2  RARELY……………………..3  NEVER………………………4 | MOST OF THE TIME...........1  SOMETIMES………………..2  RARELY……………………..3  NEVER………………………4 | MOST OF THE TIME...........1  SOMETIMES………………..2  RARELY……………………..3  NEVER……………………….4***IF RESPONDENT IS***  ***MALE*** 🡪 ***MODULE G8(A)*** |  | YES……1  NO..……2 🡪 ***MODULE G8(A)*** |
|  |  |  |  |  |  |  |  |  |
| **D** | Most senior co-wife (the person who was in the household just before you, or, if you are the senior wife, the one who married into the household after you) | **ID #** | MOST OF THE TIME...........1  SOMETIMES………………..2  RARELY……………………..3  NEVER……………………….4 | MOST OF THE TIME...........1  SOMETIMES………………..2  RARELY……………………..3  NEVER……………………….4 | MOST OF THE TIME...........1  SOMETIMES………………..2  RARELY……………………..3  NEVER……………………….4 | MOST OF THE TIME...........1  SOMETIMES………………..2  RARELY……………………..3  NEVER……………………….4 |  |  |
|  |  |  |  |  |  |  |  |  |

|  | **HOUSEHOLD ID** |  |  |  |  |  |  |
| --- | --- | --- | --- | --- | --- | --- | --- |
|  | **RESPONDENT ID** | | | | |  |  |

**MODULE G8(A): AUTONOMY IN DECISION-MAKING**

| Now I am going to read you some stories about different farmers and their situations regarding different agricultural activities. This question format is different from the rest so take your time in answering. For each I will then ask you how much you are like or not like each of these people. We would like to know if you are completely different from them, similar to them, or somewhere in between. There are no right or wrong answers to these questions.  **READ ALOUD EACH STORY, SUBSEQUENT QUESTIONs, AND RESPONSE CODES. NAMES SHOULD BE ADOPTED TO LOCAL CONTEXT AND BE MALE/FEMALE DEPENDING ON THE SEX OF THE RESPONDENT. THE ORDER OF TOPICS A-D SHOULD BE RANDOMIZED, AND WITHIN EACH TOPIC, THE ORDER OF STORIES 1-4 SHOULD BE RANDOMIZED.** | | | Are you like this person?  **CIRCLE ONE** | Are you completely the same or somewhat the same?  **CIRCLE ONE** | Are you completely different or somewhat different?  **CIRCLE ONE** |
| --- | --- | --- | --- | --- | --- |
| **STORY** | | | **G8.01** | **G8.02** | **G8.03** |
| The types of crops to grow or raise for consumption and sale in market | **A1** | *“[PERSON’S NAME] cannot grow other types of crops here for consumption and sale in market. Beans, sweet potato and maize are the only crops that grow here.”* | YES...1  NO.....2 🡪 ***G8.03*** | COMPLETELY THE SAME….1 🡪 ***A2***  SOMEWHAT THE SAME…....2 🡪 ***A2*** | COMPLETELY DIFFERENT....1  SOMEWHAT DIFFERENT.......2 |
|  | **A2** | *“[PERSON’S NAME] is a farmer and grows beans, sweet potato, and maize because her spouse, or another person or group in her community tells her she must grow these crops. She does what they tell her to do.”* | YES...1  NO.....2 🡪 ***G8.03*** | COMPLETELY THE SAME….1 🡪 ***A3***  SOMEWHAT THE SAME…....2 🡪 ***A3*** | COMPLETELY DIFFERENT....1  SOMEWHAT DIFFERENT.......2 |
|  | **A3** | *“[PERSON’S NAME] grows the crops for agricultural production that her family or community expect. She wants them to approve of her as a good farmer.”* | YES...1  NO.....2 🡪 ***G8.03*** | COMPLETELY THE SAME….1 🡪 ***A4***  SOMEWHAT THE SAME…....2 🡪 ***A4*** | COMPLETELY DIFFERENT....1  SOMEWHAT DIFFERENT.......2 |
|  | **A4** | *“[PERSON’S NAME] chooses the crops that she personally wants to grow for consumption and sale in market and thinks are best for herself and her family. She values growing these crops. If she changed her mind, she could act differently.”* | YES...1  NO.....2 🡪 ***G8.03*** | COMPLETELY THE SAME….1 🡪 ***B1***  SOMEWHAT THE SAME…....2 🡪 ***B1*** | COMPLETELY DIFFERENT....1  SOMEWHAT DIFFERENT.......2 |
| Livestock raising | **B1** | *“[PERSON’S NAME] cannot raise any livestock other than what she has. These are all that do well here.”* | YES...1  NO.....2 🡪 ***G8.03*** | COMPLETELY THE SAME….1 🡪 ***B2***  SOMEWHAT THE SAME…....2 🡪 ***B2*** | COMPLETELY DIFFERENT....1  SOMEWHAT DIFFERENT.......2 |
|  | **B2** | *“[PERSON’S NAME] raises the types of livestock she does because her spouse, or another person or group in her community tell her she must use these breeds. She does what they tell her to do.”* | YES...1  NO.....2 🡪 ***G8.03*** | COMPLETELY THE SAME….1 🡪 ***B3***  SOMEWHAT THE SAME…....2 🡪 ***B3*** | COMPLETELY DIFFERENT....1  SOMEWHAT DIFFERENT.......2 |
|  | **B3** | *“[PERSON’S NAME] raises the kinds of livestock that her family or community expect. She wants them to approve of her as a good livestock raiser.”* | YES...1  NO.....2 🡪 ***G8.03*** | COMPLETELY THE SAME….1 🡪 ***B4***  SOMEWHAT THE SAME…....2 🡪 ***B4*** | COMPLETELY DIFFERENT....1  SOMEWHAT DIFFERENT.......2 |
|  | **B4** | *“[PERSON’S NAME] chooses the types of livestock that she personally wants to raise and thinks are good for herself and her family. She values raising these types. If she changed her mind, she could act differently.”* | YES...1  NO.....2 🡪 ***G8.03*** | COMPLETELY THE SAME….1 🡪 ***C1***  SOMEWHAT THE SAME…....2 🡪 ***C1*** | COMPLETELY DIFFERENT....1  SOMEWHAT DIFFERENT.......2 |

| **READ ALOUD EACH STORY, SUBSEQUENT QUESTIONs, AND RESPONSE CODES. NAMES SHOULD BE ADOPTED TO LOCAL CONTEXT AND BE MALE/FEMALE DEPENDING ON THE SEX OF THE RESPONDENT.** | | | Are you like this person?  **CIRCLE ONE** | Are you completely the same or somewhat the same?  **CIRCLE ONE** | Are you completely different or somewhat different?  **CIRCLE ONE** |
| --- | --- | --- | --- | --- | --- |
| **STORY** | | | **G8.01** | **G8.02** | **G8.03** |
| Taking crops or livestock (incl. eggs or milk) to the market (or not) | **C1** | *“There is no alternative to how much or how little of her crops or livestock [PERSON’S NAME] can take to the market. She is taking the only possible amount.”* | YES...1  NO.....2 🡪 ***G8.03*** | COMPLETELY THE SAME….1 🡪 ***C2***  SOMEWHAT THE SAME…....2 🡪 ***C2*** | COMPLETELY DIFFERENT....1  SOMEWHAT DIFFERENT.......2 |
|  | **C2** | *“[PERSON’S NAME] takes crops and livestock to the market because her spouse, or another person or group in her community tell her she must sell them there. She does what they tell her to do.”* | YES...1  NO.....2 🡪 ***G8.03*** | COMPLETELY THE SAME….1 🡪 ***C3***  SOMEWHAT THE SAME…....2 🡪 ***C3*** | COMPLETELY DIFFERENT....1  SOMEWHAT DIFFERENT.......2 |
|  | **C3** | *“[PERSON’S NAME] takes the crops and livestock to the market that her family or community expect. She wants them to approve of her.”* | YES...1  NO.....2 🡪 ***G8.03*** | COMPLETELY THE SAME….1 🡪 ***C4***  SOMEWHAT THE SAME…....2 🡪 ***C4*** | COMPLETELY DIFFERENT....1  SOMEWHAT DIFFERENT.......2 |
|  | **C4** | *“[PERSON’S NAME] chooses to take the crops and livestock to market that she personally wants to sell there, and thinks is best for herself and her family. She values this approach to sales. If she changed her mind, she could act differently.”* | YES...1  NO.....2 🡪 ***G8.03*** | COMPLETELY THE SAME….1 🡪 ***D1***  SOMEWHAT THE SAME…....2 🡪 ***D1*** | COMPLETELY DIFFERENT....1  SOMEWHAT DIFFERENT.......2 |
| How to use income generated from agricultural and non-agricultural activities | **D1** | *“There is no alternative to how [PERSON’S NAME] uses her income. How she uses her income is determined by necessity.”* | YES...1  NO.....2 🡪 ***G8.03*** | COMPLETELY THE SAME….1 🡪 ***D2***  SOMEWHAT THE SAME…....2 🡪 ***D2*** | COMPLETELY DIFFERENT....1  SOMEWHAT DIFFERENT.......2 |
|  | **D2** | *“[PERSON’S NAME] uses her income how her spouse, or another person or group in her community tell her she must use it there. She does what they tell her to do.”* | YES...1  NO.....2 🡪 ***G8.03*** | COMPLETELY THE SAME….1 🡪 ***D3***  SOMEWHAT THE SAME…....2 🡪 ***D3*** | COMPLETELY DIFFERENT....1  SOMEWHAT DIFFERENT.......2 |
|  | **D3** | *“[PERSON’S NAME] uses her income in the way that her family or community expect. She wants them to approve of her.”* | YES...1  NO.....2 🡪 ***G8.03*** | COMPLETELY THE SAME….1 🡪 ***D4***  SOMEWHAT THE SAME…....2 🡪 ***D4*** | COMPLETELY DIFFERENT....1  SOMEWHAT DIFFERENT.......2 |
|  | **D4** | *“[PERSON’S NAME] chooses to use her income how she personally wants to, and thinks is best for herself and her family. She values using her income in this way. If she changed her mind, she could act differently.”* | YES...1  NO.....2 🡪 ***G8.03*** | COMPLETELY THE SAME...1🡪***G8.04***  SOMEWHAT THE SAME….2 🡪***G8.04*** | COMPLETELY DIFFERENT....1  SOMEWHAT DIFFERENT.......2 |

**MODULE G8(B): NEW GENERAL SELF-EFFICACY SCALE**

| Now I’m going to ask you some questions about different feelings you might have. Please listen to each of the following statements. Think about how each statement relates to your life, and then tell me how much you agree or disagree with the statement on a scale of 1 to 5, where 1 means you “strongly disagree” and 5 means you “strongly agree.” (**Note: Randomize order of statements**) | | |
| --- | --- | --- |
| **Statements** | | **G8.04** |
| **A** | I will be able to achieve most of the goals that I have set for myself. | STRONGLY DISAGREE 1  DISAGREE 2  NEITHER AGREE NOR DISAGREE 3  AGREE 4  STRONGLY AGREE 5 |
| **B** | When facing difficult tasks, I am certain that I will accomplish them. | STRONGLY DISAGREE 1  DISAGREE 2  NEITHER AGREE NOR DISAGREE 3  AGREE 4  STRONGLY AGREE 5 |
| **C** | In general, I think that I can obtain outcomes that are important to me. | STRONGLY DISAGREE 1  DISAGREE 2  NEITHER AGREE NOR DISAGREE 3  AGREE 4  STRONGLY AGREE 5 |
| **D** | I believe I can succeed at most any endeavor to which I set my mind | STRONGLY DISAGREE 1  DISAGREE 2  NEITHER AGREE NOR DISAGREE 3  AGREE 4  STRONGLY AGREE 5 |
| **E** | I will be able to successfully overcome many challenges. | STRONGLY DISAGREE 1  DISAGREE 2  NEITHER AGREE NOR DISAGREE 3  AGREE 4  STRONGLY AGREE 5 |
| **F** | I am confident that I can perform effectively on many different tasks. | STRONGLY DISAGREE 1  DISAGREE 2  NEITHER AGREE NOR DISAGREE 3  AGREE 4  STRONGLY AGREE 5 |
| **G** | Compared to other people, I can do most tasks very well. | STRONGLY DISAGREE 1  DISAGREE 2  NEITHER AGREE NOR DISAGREE 3  AGREE 4  STRONGLY AGREE 5 |
| **H** | Even when things are tough, I can perform quite well. | STRONGLY DISAGREE 1  DISAGREE 2  NEITHER AGREE NOR DISAGREE 3  AGREE 4  STRONGLY AGREE 5 |

**MODULE G8(C): LIFE SATISFACTION**

| The following questions ask how satisfied you feel with your life as a whole, on a scale from 1 to 5, where 1 means you feel “very dissatisfied” and 5 means you feel “very satisfied.” | | |
| --- | --- | --- |
|  | **Statements** | **G8.05** |
| **A** | Overall, how satisfied are you with life as a whole these days? | VERY DISSATISFIED 1  DISSATISFIED 2  NEITHER SATISFIED NOR DISSATISFIED 3  SATISFIED 4  VERY SATISFIED 5 |
| **B** | Overall, how satisfied with your life were you 5 years ago? | VERY DISSATISFIED 1  DISSATISFIED 2  NEITHER SATISFIED NOR DISSATISFIED 3  SATISFIED 4  VERY SATISFIED 5 |
| **C** | As your best guess, overall how satisfied with your life do you expect to feel 5 years from today? | VERY DISSATISFIED 1  DISSATISFIED 2  NEITHER SATISFIED NOR DISSATISFIED 3  SATISFIED 4  VERY SATISFIED 5 |

| **HOUSEHOLD ID** |  |  |  |  |  |  |
| --- | --- | --- | --- | --- | --- | --- |
| **RESPONDENT ID** | | | | |  |  |

**MODULE G9. Attitudes about Domestic Violence**

| Now I would like to ask about your opinion on the following issues. Please keep in mind that I am not asking about your personal experience or whether the following scenarios have happened to you. I would only like to know whether you think the following issues are acceptable. | | In your opinion, is a husband justified in hitting or beating his wife in the following situations? |
| --- | --- | --- |
| **SITUATION** | | **G9.01** |
| **A** | If she goes out without telling him? | YES 1  NO 2  DON’T KNOW 97 |
| **B** | If she neglects the children? | YES 1  NO 2  DON’T KNOW 97 |
| **C** | If she argues with him? | YES 1  NO 2  DON’T KNOW 97 |
| **D** | If she refuses to have sex with him? | YES 1  NO 2  DON’T KNOW 97 |
| **E** | If she burns the food? | YES 1  NO 2  DON’T KNOW 97 |

# Appendix D: Glossary of WEAI terms

**Abbreviated Women’s Empowerment in Agriculture Index (A-WEAI)**: An aggregate index that measures women’s empowerment and inclusion in the agriculture sector that is a shorter version of the original WEAI; A-WEAI is based on interviews of the primary male and female decision-maker in each household, is reported at the country or regional level, and is comprised of two sub-indices, the Five Domains of Empowerment subindex (5DE) and Gender Parity Index (GPI)

**Adequacy score**: The weighted average proportion of indicators of WEAI, A-WEAI, or pro-WEAI in which a respondent is adequate; also called empowerment score

**Adequate**: When a respondent achieves a certain threshold in an indicator of WEAI, A-WEAI, or pro-WEAI, they are considered adequate in that indicator and assigned a value of one in the binary indicator when calculating the index

**Access to and decisions on credit**: One of 10 indicators of WEAI; one of six indicators of A-WEAI; a respondent is considered adequate when they participate in decisions about at least one source of credit used by their household in the past year or belong to a household that did not use credit in the past year but could have if they wanted to

**Access to and decisions on financial services**: One of 12 indicators of pro-WEAI; a respondent is considered adequate when they participate in decisions about at least one source of credit used by their household in the past year, belong to a household that did not use credit in the past year but could have if they wanted to, or have sole or joint access to a financial account

**Achievements**: One of the three interrelated dimensions of empowerment according to Naila Kabeer’s (1999) definition; achievement of well-being related outcomes such as income, health, and nutrition

**Agency**: One of the three interrelated dimensions of empowerment according to Naila Kabeer’s (1999) definition; the capacity to define one’s own goals and make strategic choices in pursuit of these goals, particularly in a context where this ability was previously denied; all forms of WEAI focus on measuring agency

**Alkire-Foster method**: An axiomatic and counting-based approach designed for measuring multidimensional poverty that was used to develop the measure of disempowerment used in WEAI, A-WEAI, and pro-WEAI

**Attitudes about intimate partner violence against women**: One of 12 indicators of pro-WEAI; a respondent is considered adequate when believe that a husband is not justified in hitting or beating his wife in all of five scenarios

**Autonomy in income**: One of 12 indicators of pro-WEAI; a respondent is considered adequate when they are more motivated by their own values than by coercion or fear of others’ disapproval in how to use income generated from agricultural and non-agricultural activities, calculated as when their Relative Autonomy Index is greater than or equal to one

**Autonomy in production**: One of 10 indicators of WEAI; a respondent is considered adequate when they are more motivated by their own values than by coercion or fear of others’ disapproval in the types of crops to grow or raise, livestock raising, or taking crops or livestock to market, calculated as when their Relative Autonomy Index is greater than or equal to one

**Censored headcount ratio**: The proportion of respondents who are disempowered and inadequate in a given indicator

**Censored inadequacy score**: The weighted average proportion of indicators of WEAI, A-WEAI, or pro-WEAI in which a respondent is inadequate, set to equal the disempowerment cutoff if the individual has not achieved empowerment

**Collective agency**: Power with, or the power gained from acting together with others; one of three domains of pro-WEAI; one of the generative types of power described by Rowlands (1997) and Ibrahim and Alkire (2007)

**Contribution to disempowerment**: How much each indicator contributes to disempowerment among respondents who have not achieved empowerment; calculated as the indicator weight multiplied by the indicator censored headcount ratio

**Control over use of income**: One of 12 indicators of pro-WEAI; one of 10 indicators of WEAI; one of six indicators of A-WEAI; for pro-WEAI, a respondent is considered adequate when they have input in decisions about how to use both income and output from all agricultural activities they participate in, and have input in decisions about how to use income from all non-agricultural activities they participate in; for WEAI and A-WEAI, a respondent is considered adequate when they have input in decisions about how to use income from at least one agricultural or non-agricultural activity

**Decomposition**: Breaking down the 5DE or 3DE by population subgroups, such as gender, age, geographic location, ethnicity, etc., for which the sample is representative

**Dimensional breakdown**: Breaking down the 5DE and 3DE by domain and/or indicator to look at how many respondents were adequate in each indicator or how much each domain and/or indicator contributed to disempowerment among respondents who have not achieved empowerment

**Disempowerment cutoff**: The percentage of weighted indicators in which a respondent must be inadequate to be considered disempowered according to WEAI, A-WEAI, or pro-WEAI

**Disempowerment headcount ratio**: The proportion of respondents who have not achieved empowerment

**Disempowerment score**: The inverse of the 3DE or 5DE; an aggregate index that reflects the percentage of women who are disempowered and the intensity of disempowerment among women who have not achieved empowerment

**Domain**: A thematic area of empowerment measured by WEAI, A-WEAI, or pro-WEAI; the five domains of WEAI and A-WEAI are production, resources, income, leadership, and time; the three domains of pro-WEAI are intrinsic, instrumental, and collective agency

**Dual-adult household (DHH)**: A household that includes both a male and female adult

**Empowered**: For WEAI and A-WEAI, when a respondent has achieved adequacy in 80% of more of the weighted indicators; for pro-WEAI, when a respondent has achieved adequacy in 75% or more of the weighted indicators

**Empowerment**: The process by which people expand their ability to make strategic life choices, particularly in context in which this ability has been denied to them (Kabeer 1999)

**Empowerment cutoff**: The percentage of weighted indicators in which a respondent must achieve adequacy to be considered empowered according to WEAI, A-WEAI, or pro-WEAI

**Empowerment gap**: The average percentage difference in empowerment between men and women in households that have not achieved gender parity

**Empowerment score**: The weighted average proportion of indicators of WEAI, A-WEAI, or pro-WEAI in which a respondent is adequate; also called adequacy score

**Female-adult-only household (FHH)**: A households that includes a female adult but does not include a male adult

**Five Domains of Empowerment Index (5DE)**: One of the two sub-indices, weighted 90%, that make up WEAI and A-WEAI; an aggregate index that reflects women’s achievements across five domains (production, resources, income, leadership, and time); reflects the percentage of women who are empowered and the intensity of disempowerment among women who have not achieved empowerment

**Gender, Agriculture, and Assets Project, Phase 2 (GAAP2)**: A project that aims to adapt and validate a measure of women’s empowerment that agricultural development projects can use to diagnose key areas of women’s and men’s disempowerment and design appropriate strategies to address deficiencies and monitor project outcomes related to women’s empowerment; this project is developing the project-level Women’s Empowerment in Agriculture Index (pro-WEAI); GAAP2 is led by the International Food Policy Research Institute, funded by the Bill and Melinda Gates Foundation, the United States Agency for International Development, and the CGIAR Research Program on Agriculture for Nutrition and Health, and includes collaborative portfolio of 13 agricultural development projects in Africa and South Asia that are piloting and helping to develop pro-WEAI

**Gender parity**: When a woman is empowered or has achieved adequacy in at least as many indicators as the male in her household

**Gender Parity Index (GPI)**: One of the two sub-indices, weighted 10%, that make up the WEAI, A-WEAI, and pro-WEAI; an aggregate index that reflects gender parity; reflects the percentage of women in dual-adult households who are empowered or as empowered as the male in their household, and the average percentage difference in empowerment between men and women in households that have not achieved gender parity

**Group membership**: One of 12 indicators of pro-WEAI; one of 10 indicators of WEAI; one of six indicators of A-WEAI; for pro-WEAI, WEAI, and A-WEAI, a respondent is considered adequate when they are an active member of at least one community group

**Inadequacy score**: The weighted average proportion of indicators of WEAI, A-WEAI, or pro-WEAI in which a respondent is inadequate

**Inadequate**: When a respondent has not yet achieved a certain threshold in an indicator of WEAI, A-WEAI, or pro-WEAI, they are considered inadequate in that indicator and assigned a value of zero in the binary indicator when calculating the index

**Indicator**: A dimension of empowerment measured by WEAI, A-WEAI, and pro-WEAI; there are 10 indicators of WEAI and A-WEAI and 12 indicators of pro-WEAI; each is a binary indicator that equals one when a respondent achieves a certain threshold in that dimension

**Input in productive decisions**: One of 12 indicators of pro-WEAI; one of 10 indicators of WEAI; one of six indicators of A-WEAI; for pro-WEAI, a respondent is considered adequate when, for all of the agricultural activities they participate in, they make decisions solely, make decisions jointly and have at least some input in the decisions, or feel that they could make decisions if they wanted to at least a medium extent; for WEAI and A-WEAI, a respondent is considered adequate when, for at least one agricultural activity, they make decisions solely, make decisions jointly and have at least some input in the decisions, or feel that they could make decisions if they wanted to at least a medium extent

**Instrumental agency**: Power to, or a person’s ability to make decisions in their own best interest; one of three domains of pro-WEAI; one of the generative types of power described by Rowlands (1997) and Ibrahim and Alkire (2007)

**Intensity of disempowerment**: The average proportion of indicators in which respondents who have not achieved empowerment are inadequate

**Intrinsic agency**: Power within, or a person’s internal voice, self-respect, and self-confidence; one of three domains of pro-WEAI; one of the generative types of power described by Rowlands (1997) and Ibrahim and Alkire (2007)

**Leisure**: One of 10 indicators of WEAI; a respondent is considered adequate when they rank their level of satisfaction with their time available for a set of leisure activities as an average of five or greater on a scale from one to 10

**Membership in influential groups**: One of 12 indicators of pro-WEAI; a respondent is considered adequate when they are an active member of at least one group that they report can influence the community to at least a medium extent

**New General Self-Efficacy Scale (NGSE)**: A validated scale to measure self-efficacy, or a person’s capabilities and ability to reach their goals (Chen, Gully and Eden 2001)

**Ownership of assets**: One of 10 indicators of WEAI; one of six indicators of A-WEAI; a respondent is considered adequate when they solely or jointly own at least two small assets or one large asset

**Ownership of land and other assets**: One of 12 indicators of pro-WEAI; a respondent is considered adequate when they solely or jointly own at least three small assets, two large assets, or land

**Project-level Women’s Empowerment in Agriculture Index (pro-WEAI)**: An aggregate index that measures empowerment, agency, and inclusion of women in agricultural development projects; pro-WEAI is based on interviews of the primary adult male and female decision-makers or project participants in each household, is reported at the project level, and is comprised of two sub-indices, 3DE and GPI

**Proportional contribution to disempowerment**: How much each indicator contributes to disempowerment among respondents who have not achieved empowerment, as a percentage of the total disempowerment score; calculated as the indicator weight multiplied by the indicator censored headcount ratio divided by the total disempowerment score multiplied by 100

**Purchase, sale, or transfer of assets**: One of 10 indicators of WEAI; a respondent is considered adequate when they participate in decisions about the purchase, sale, or transfer about at least one agricultural asset

**Redundancy**: For indicators A and B, the proportion of respondents inadequate in indicator A who are simultaneously inadequate in indicators B, where A is the indicator in which fewer respondents are inadequate

**Relative Autonomy Index (RAI)**: A measure of the internal and external motivations that determine a person’s decisions, based on self-determination theory (Ryan and Deci 2000)

**Resources**: One of the three interrelated dimensions of empowerment according to Naila Kabeer’s (1999) definition; access and future claims to the material, human, and social resources that serve to enhance one’s ability to exercise choice

**Respect among household members**: One of 12 indicators of pro-WEAI; a respondent is considered adequate when they report that they respect, are respected by, trust, and feel comfortable disagreeing with their spouse, the other respondent in the household, or another adult household member

**Self-efficacy**: One of 12 indicators of pro-WEAI; a respondent is considered adequate when they have a score of 32 or greater on the New General Self-Efficacy Scale, meaning that they answer “agree” or greater on average to a set of self-efficacy questions

**Speaking in public**: One of 10 indicators of WEAI; a respondent is considered adequate when they rate their comfort speaking in public as an average of at least two on a scale of one to five across three situations: to help decide on infrastructure, to ensure proper payment of wages, and to promote misbehavior of authorities or elected officials

**Three Domains of Empowerment Index (3DE)**: One of the two sub-indices, weighted 90%, that make up pro-WEAI; an aggregate index that reflects women’s achievements across 12 indicators; reflects the percentage of women who are empowered and the intensity of disempowerment among women who have not achieved empowerment

**Uncensored headcount ratio**: The proportion of respondents who are inadequate in a given indicator, regardless of their empowerment status

**Visiting important locations**: One of 12 indicators of pro-WEAI; a respondent is considered adequate when they visit at least two of three locations in their community at least once per week – city, market, and family/relative – or visit a health facility or public meeting at least once per month

**WEAI Resource Center**: Website that provides background, resources, news, publications, and datasets related to all versions of WEAI; weai.ifpri.info

**Weight**: The proportional weight given to each indicator of when calculating empowerment; in pro-WEAI, each indicator is weighted 1/12

**Women’s Empowerment in Agriculture Index (WEAI)**: An aggregate index that measures women’s empowerment and inclusion in the agriculture sector; WEAI is based on interviews of the primary male and female decision-maker in each household, is reported at the country or regional level, and is comprised of two sub-indices, 5DE and GPI

**Work balance**: One of 12 indicators of pro-WEAI; a respondent is considered adequate when they work less than 10.5 hours per day, where workload is calculated as the time spent on work as a primary activity plus half of the time spent on childcare as a secondary activity

**Workload**: One of 10 indicators of WEAI; one of six indicators of A-WEAI; a respondent is considered adequate when they work less than 10.5 hours per day
